# Supplementary material for: Regional multidisciplinary team intervention programme to improve colorectal cancer outcomes: study protocol for the Yorkshire Cancer Research Bowel Cancer Improvement Programme (YCR BCIP)
Source: BMJ Open. 2019 Nov 26;9(11):e030618. doi: 10.1136/bmjopen-2019-030618 (PMC6886907; doi:10.1136/bmjopen-2019-030618)
Supplement: Author's manuscript [file bmjopen-2019-030618.draft_revisions.pdf]

BMJ Open

BMJ Open is committed to open peer review. As part of this commitment we make the peer review history of every article we publish publicly available.

When an article is published we post the peer reviewers' comments and the authors' responses online. We also post the versions of the paper that were used during peer review. These are the versions that the peer review comments apply to.

The versions of the paper that follow are the versions that were submitted during the peer review process. They are not the versions of record or the final published versions. They should not be cited or distributed as the published version of this manuscript.

BMJ Open is an open access journal and the full, final, typeset and author-corrected version of record of the manuscript is available on our site with no access controls, subscription charges or pay-per-view fees (http://bmjopen.bmj.com).

If you have any questions on BMJ Open's open peer review process please email info.bmjopen@bmj.com

BMJ Open

The Yorkshire Cancer Research Bowel Cancer Improvement Programme (YCR BCIP): study protocol

Journal:	*BMJ Open*
Manuscript ID	bmjopen-2019-030618
Article Type:	Protocol
Date Submitted by the Author:	22-Mar-2019
Complete List of Authors:	Taylor, John; Leeds Institute of Cancer and Pathology, Section of Epidemiology and Biostatistics, University of Leeds<br>Wright, Penny; Leeds Institute of Cancer and Pathology, Section of Patient Centred Outcome Research, University of Leeds<br>Rossington, Hannah; Leeds Institute of Cancer and Pathology, Section of Epidemiology and Biostatistics, University of Leeds<br>Mara, Jackie; Leeds Institute of Cancer and Pathology, Section of Epidemiology and Biostatistics, University of Leeds<br>Glover, Amy; Leeds Institute of Cancer and Pathology, Section of Pathology and Tumour Biology, University of Leeds<br>West, Nick; Leeds Institute of Cancer and Pathology, Section of Pathology and Tumour Biology, University of Leeds<br>Morris, Eva; Leeds Institute of Cancer and Pathology, Section of Epidemiology and Biostatistics, University of Leeds<br>Quirke, Phillip; Leeds Institute of Cancer and Pathology, Section of Pathology and Tumour Biology, University of Leeds
Keywords:	Quality in health care < HEALTH SERVICES ADMINISTRATION & MANAGEMENT, Gastrointestinal tumours < ONCOLOGY, PUBLIC HEALTH, STATISTICS & RESEARCH METHODS, EPIDEMIOLOGY

SCHOLARONE™
Manuscripts

The Yorkshire Cancer Research Bowel Cancer Improvement Programme (YCR BCIP): study protocol

J.C. Taylor[1*], P. Wright[2], H.L. Rossington[1], J. Mara[1], A. Glover[3], N.P. West[3], E.J.A. Morris[1], P. Quirke[3] on behalf of YCR BCIP Study Group.

1. Cancer Epidemiology Group, Section of Epidemiology and Biostatistics, Leeds Institute of Cancer & Pathology and Leeds Institute for Data Analytics, University of Leeds, Leeds, UK
2. Section of Patient Centred Outcome Research, Leeds Institute of Cancer & Pathology, University of Leeds, Leeds, UK
3. Section of Pathology, Leeds Institute of Medical Research at St. James's, University of Leeds, Leeds, UK

Corresponding author:

J. C. Taylor

Leeds Institute for Data Analytics

Worsley Building

University of Leeds

LS2 9NL, Leeds, UK

j.c.taylor@leeds.ac.uk

For peer review only

Abstract

Introduction

Although colorectal cancer outcomes in England are improving they remain poorer than many comparable countries. Yorkshire Cancer Research have, therefore, established a Bowel Cancer Improvement Programme (YCR BCIP) to improve colorectal cancer outcomes within the Yorkshire and Humber, a region representative of the nation in these terms. It aims to do this by quantifying variation in practice, engaging with the colorectal multidisciplinary teams (MDTs) to understand this and instigating interventions to minimise it and improve outcomes.

Methods and analysis

Initially, routine health datasets will be used to quantify variation in the demographics, management and outcomes of patients across the Yorkshire and the Humber region and results presented to MDTs. The YCR BCIP is seeking to supplement these existing data with patient reported health-related quality of life information (PROMs) and tissue sample analysis. Specialty groups (surgery, radiology, pathology, clinical oncology, medical oncology, clinical nurse specialists and anaesthetics) have been established to provide oversight and direction for their clinical area within the programme, to review data and analysis and to develop appropriate educational initiatives.

Ethics and dissemination

The YCR BCIP is seeking to address the variation in practice to significantly improve colorectal cancer outcomes across the Yorkshire and Humber region. PROMs and tissue sample collection and analysis will help to capture the information required to fully assess care in the region. Engagement of the region's MDTs with their data will lead to a range of educational initiatives, studies and clinical audits that aim to optimise practice across the region.

For peer review only

Stengths and limitations of this study

- Evaluating variation in the management and outcomes of colorectal cancer patients using routinely collected clinical datasets and new information on aspects of care not currently quantifiable through existing datasets.
- Providing regional colorectal MDTs with data and, with their input, develop interventions to minimise any variation seen in order to optimise outcomes.
- Facilitating implementation of guidelines such as routine screening for Lynch syndrome and deficient mismatch repair status.

For peer review only

For peer review only - http://bmjopen.bmj.com/site/about/guidelines.xhtml

Introduction

Colorectal cancer is a common disease in the UK with over 41,000 people diagnosed each year and 16,000 people dying from it. Although survival rates have improved over time, they continue to lag behind those attained by many economic neighbours[1] and so developing interventions to improve outcomes is a priority.

Some potential explanations for these survival differences include variability in the quality of care and access to services. For example, local recurrence and survival rates in rectal cancer are related to the quality of the surgical resection[2] so improving surgical quality is likely to improve outcomes. Likewise, ensuring gold standard diagnostic, staging and multidisciplinary care across all aspects of the patient pathway have a demonstrable impact on outcomes[3] while screening for Lynch syndrome and deficient mismatch repair will enable more effective managemement of patients.

Other countries have acknowledged these relationships and taken steps to improve standards. For instance, in the early 1990s, Norway recognised there was huge variation in local recurrence rates depending on which surgeon operated. In response, they initiated a training programme to quantify variation and improve the quality of surgery. This strategy was a success, reducing local recurrence and improving overall survival[4]. As a result similar programmes in other countries, including the UK [3, 5-9] were established with equally positive outcomes including lower postoperative mortality and better survival, lower permanent stoma and local recurrence rates, better preoperative staging, improved selection of patients for non-surgical treatment and the need for less emergency surgery.

Whilst these programmes have undoubtedly had a positive impact, variations in management remain and particularly so in the UK. Here, although outcomes have improved, the gap to rates attained in comparable countries[10] have not closed. Better understanding of what is driving these variations will help to target interventions to improve care.

Yorkshire Cancer Research is a charity whose aim is to ensure that more people of Yorkshire avoid, survive and cope with cancer and, by 2025, they intend to have reduced cancer deaths in the region by 2,000 per year. As part of their strategy to achieve this they have recently funded the Bowel Cancer Improvement Programme (YCR BCIP). This initiative

For peer review only - http://bmjopen.bmj.com/site/about/guidelines.xhtml

builds upon the experience gained from several English National programmes [8, 9] and extends the approach of those successful programmes completed in Scandinavia [3, 5, 6]. It centres on the collection and analysis of robust colorectal cancer data to quantify practice across the region, ensuring the multidisciplinary teams (MDTs) managing the disease engage with these data and agree areas for improvement. Educational interventions or other strategies can then be implemented to ensure optimal practice is achieved.

The aims of the YCRBCIP study are to:

1. Quantify and report the variation in the demographics, management and outcomes of the region's colorectal cancer patients using
    a. routinely collected clinical datasets,
    b. new information on aspects of care not currently quantifiable through existing datasets (e.g. Patient Reported Outcome Measures (PROMs), the molecular subtyping of the disease and the quality of radiology, pathology and surgery).
2. Where comparative data are available, to determine how outcomes from the Yorkshire and Humber region compare to the rest of England.
3. To provide the Yorkshire and Humber region colorectal MDTs with these data and, with their input, develop interventions to minimise any variation seen in order to optimise outcomes
4. Facilitate routine screening for Lynch syndrome and deficient mismatch repair status.
5. Evaluate improvement in outcomes over the study period.

For peer review only

Methods and analysis

Setting

The Yorkshire and Humber region of the UK has a population of approximately 5.7 million with around 3,300 colorectal cancer diagnoses a year, amounting to just over 10% of English diagnoses. Patients diagnosed with colorectal cancer in the region are managed by 16 different MDTs. These operate across 14 National Health Service (NHS) Trusts which provide health services for particular geographical areas of the region which exhibit a range of workloads, ranging between approximately 120-390 cases a year (4%-12% of the regional workload). Patients within the geographical areas covered by each Trust exhibit a variable range of demographic features such as socioeconomic deprivation and comorbidity [11].

Programme design overview

YCR BCIP will interrogate routinely collected NHS data and, subject to patient consent, additional data collected specifically for the programme. Baseline performance outcomes for each MDT from across the region will be assessed. The knowledge will be fed back to each MDT and a programme of educational interventions developed based on the performance outcomes. The process will be iterative as outlined in Figure 1. The programme will run from 1st April 2016 until 31 March 2021.

Clinical specialities

Following a regional meeting of MDT representatives to discuss, plan and agree to programme participation held in June 2016, six specialty groups were initially established. The remit of these groups is to provide oversight and direction for their clinical specialty within the programme, to review data and analyses and to develop appropriate educational initiatives. The six groups focused on the main clinical disciplines involved in the MDT; surgery, radiology, pathology, clinical oncology, medical oncology, and specialist nursing. Group membership was drawn from all of the region's MDTs and an individual established from each to to act as a coordinator between the research team and MDT. The need for a seperate anaesthetics workstream was subsequently identified and introduced.

Data collection

Although broad ranging, it was recognised that the routine colorectal cancer data available is not sufficient to quantify all aspects of care robustly. Whilst routine data provides good

For peer review only - http://bmjopen.bmj.com/site/about/guidelines.xhtml

information available on 'hard' outcomes such as which operation was undertaken or survival time, there is a lack of information on what this really means to the individual concerned and the quality of that survival. This precludes the production of the rich intelligence the MDTs want to fully understand variation in care. Therefore data collected in YCR BCIP will come from two main sources:

1. routinely collected NHS datasets,
2. additional data collected subject to patient written informed consent;
    a. PROMs data, collected directly from patients on health-related quality of life at both the time of diagnosis (and before primary treatment if possible) and again at 12 months post diagnosis.
    b. Molecular testing of excess pre-treatment diagnostic biopsy tissue, excess tissue following surgical resection, (both tumour and tumour-associated normal mucosal tissue), and excess tissue following the biopsy or resection of distant metastases.
    c. Screening for Lynch syndrome and deficient mismatch repair on routinely diagnosed bowel cancers.

Routine NHS data

The YCR BCIP seeks to make maximum use of routine NHS datasets and these are brought together in the UK Bowel Cancer Intelligence Hub[12] where data from the National Cancer Registry and Analysis Service (NCRAS)[13] are linked to other datasets relevant to colorectal cancer to provide the richest data possible and enable analysis of the full cancer pathway. These include, but are not limited to: Hospital Episode Statistics, Radiotherapy Dataset, Systematic Anti-Cancer Therapy Datatset and Routes to Diagnosis. This will provide both regional data (around 3,300 cases per year) and comparative national data (around 28,700 cases per year) on patient and tumour characteristics, treatment choices, diagnosis pathways and patient outcomes. This data will cover ten years preceding the start of the programme to provide a baseline and be routinely updated to assess changes throughout it.

Data collected requiring patient written informed consent

Participants

All MDTs in the region have been invited to participate in this element of the YCR BCIP. Every colorectal cancer patient (ICD10 C18-C20) is eligible if they are considered suitable for

For peer review only - http://bmjopen.bmj.com/site/about/guidelines.xhtml

treatment, English language literate, aged at least 18 years old and with capacity to give informed consent. Participants will be asked to consent to the study prior to commencement of primary treatment or following an emergency intervention.

Sample size

Based on the number of diagnoses per year, there is potential to consent approximately 8,250 patients from sites across the region over a 30 month recruitment period. However due to eligibility restrictions, consent by MDT and by individuals, administrative/practical issues and staggered site recruitment start dates, the participation rate is likely to be less. Using estimates based on previous studies [14, 15] the consent rate is estimated to be around 42% and the an attrition rate of approximately 35% at the 12 months follow-up point, giving a sample size of at least 1200. This will provide MDTs with a comprehensive descriptive profile of their patients in terms of quality of life outcomes; more complex analyses will be undertaken if sufficient numbers are accrued.

Patient recruitment

This research project is adopted by the NIHR Yorkshire and Humber Clinical Research Network and therefore recruitment will be undertaken across the region by network research and clinical staff working collaboratively. Recruitment will run over a 30 month period.

Eligible patients will be identified and approached in two ways.

1. Identified via the MDT and informed about the study by consultant letter sent out with their appointment letter for the primary pre-assessment clinic visit . Where possible, patients will be approached about the study at this clinic appointment. Patients missed at this appointment will be contacted at the earliest convenient time point and informed about the study.

2. Identified by their NHS clinical team if they present as an acute admission (for example with a bowel obstruction) and informed about the study by their clinical team following the emergency intervention (e.g. surgery).

Interested patients will be provided with full study information (written and verbal). Written consent may be taken at the time of this approach but patients will be given up to a week to think about study participation. Patients who wish to join the study will be asked to read, complete and sign a consent form, including their contact details name, address and/or

For peer review only - http://bmjopen.bmj.com/site/about/guidelines.xhtml

email address. The person taking consent will also record the patient's date of birth, NHS number and gender.

Patient participation

Participants will have the option of completing the PROMs either online (ePROMs) using the University of Leeds secure questionnaire administration system QTool [16], accessed via the study website, (www.YCRBCIP.leeds.ac.uk) or on paper (pPROMs) with a pre-paid return envelope. They will be asked to complete the survey as soon as possible after consenting, this may be completed while attending hospital at the time of consent or later at home. Just prior to the 12 month follow-up the patient status will be checked via NCRAS to confirm that the patient is still alive. Patients will then be sent an email/letter 12 months post-diagnosis by the YCR BCIP research team, inviting them to complete PROMs again (on paper or online according to patient preference). At both time points reminder letters or emails will be sent at two weeks and followed up two weeks later (if no response) with the survey being resent with the reminder letter. The process is outlined in Figure 2. Tissue samples removed at surgery or biopsy which are surplus to routine clinical requirements will be utilised by the research team for upfront testing of novel biomarkers.

Management of consent and follow-up procedures

Participant recruitment will be undertaken by each Trust involved in the study. Each research network site will allocate a study ID for potential participants. They will use a University of Leeds secure electronic transfer system every two weeks to inform the YCR BCIP research team of all recruitment activity. This will include the consented patients contact details, date of birth and NHS number to allow for follow-up and to ensure tissue blocks are appropriately labelled for tracking and data linkage. All subsequent participant contact will be undertaken by the central YCR BCIP team.

Patient Reported Outcome Measures (PROMs)

Participants will be asked to complete a range of validated generic, cancer-specific and colorectal cancer-specific PROMs in addition to socio-demographic characteristics. Content has been informed by: clinical relevance, opinion of service users, overall length and participant burden[17], reviews of questionnaires measuring quality of life in colorectal cancer patients[18-22] and UK recommendations for the core outcomes set for trials in colorectal cancer surgery[23].

For peer review only - http://bmjopen.bmj.com/site/about/guidelines.xhtml

The survey comprises four sections at baseline and five sections at follow-up. The same PROMs are included at both time points. In addition, at the second time point (12-months post diagnosis) participants will be asked to complete questions about the financial costs of cancer. The time taken to complete the survey is estimated is about 30-35 minutes.

Section One: Your overall health and quality of life (both time points)

- European Organisation for Research and Treatment of Cancer (EORTC) Quality of Life Questionnaire QLQ-C30, the colorectal cancer specific module and items from the prostate, endometrial and cervical cancer modules [24-28]
- EuroQol Group EQ-5D-5L [29]

Section Two: Your everyday life and well-being (both time points)

- Social Difficulties Inventory (SDI-21) [30, 31]
- The Short Warwick-Edinburgh Mental Well-being Scale (SWEMWBS) [32, 33]

Section Three: Manging your health (both time points)

- Self-efficacy for Managing Chronic Disease [34]
- The Brief Illness Perceptions Questionnaire (B-IPQ) [35]

Section Four: The financial cost of cancer (Time 2 only)

- A questionnaire developed in-house based on one used in the ePOCS study[36].

Section Four: Questions about you (Time 1) and Section Five: Questions about you (Time 2)

- Self-report socio-demographic and clinical details

Tumour and normal mucosal tissue samples

The tissue samples to be collected for this study are:

1. excess pre-treatment, diagnostic biopsy tumour tissue
2. excess tissue following surgical resection (tumour and tumour-associated normal mucosal tissue)
3. excess tumour tissue following the biopsy or resection of distant metastases

The tissue samples will be formalin-fixed and paraffin wax-embedded as per the routine histopathology procedures at the patients' hospital. Tissue blocks will be used for any routine clinical diagnostic procedures required by the hospital before being sent to Pathology at the University of Leeds, and will be available to return to the hospital for further clinical testing if required.

For peer review only - http://bmjopen.bmj.com/site/about/guidelines.xhtml

YCR BCIP will test for novel or rare potentially treatable targets via a range of molecular techniques. The immunohistochemical markers of interest include, HER2, PTEN, PD-1 and PD-L1, amphiregulin and epiregulin, and immune system markers CD3 and CD8. The study will also undertake phenotype analysis by using high-resolution scanned images of tumours using novel algorithms to identify improved prognostic and predictive markers of outcome.

Next Generation Sequencing and/or pyrosequencing will be performed on any extracted DNA to identify tumours with specific gene hotspot mutations including, KRAS, NRAS, BRAF, EGFR and PIK3CA. Other molecular markers linked to causation, outcome and response to therapy will also be investigated e.g. bacterial toxin carriage or fusobacterium nucleatum. Analysis of RNA expression levels of receptor ligands such as amphiregulin and epiregulin, and HER2 and HER3 may also be performed.

Patients are asked to consent to their clinical team being informed in the event that clinically relevant laboratory results are found. This includes the possibility of hereditary conditions identified through tumour testing e.g. Lynch syndrome or deficient mismatch repair status. These will be confirmed through routine NHS Clinical Genetics testing after counselling following referral from the local clinical teams with no germline testing taking place through the programme.

Data analysis

Baseline assessments of care in the region are to be performed on individuals diagnosed with colorectal cancer (ICD10 C18-C20) in Yorkshire and the Humber, enabling comparison between teams in the region and with national data[11]. Initially this includes using descriptive analysis and statistical methods such as survival analysis and funnel plots [37] comparing the following data:

- demographic characteristics (age, gender, socioeconomic status (income domain of the Index of Multiple Deprivation [IMD] 2010) and Charlson comorbidity level);
- tumour characteristics (site and stage of disease);
- surgical management (major resection, 18-month permanent stoma following major resection, laparoscopic surgery for major resections and abdominoperineal excision rates);
- outcomes (30-day postoperative mortality and 3-year survival, PROMs);

For peer review only - http://bmjopen.bmj.com/site/about/guidelines.xhtml

- use of neoadjuvant radiotherapy and timing of the proceeding surgery;
- use of adjuvant chemotherapy.

Some analyses will be rerun periodically over the course of the programme to evaluate the impact on outcomes of specific educational interventions.

The routinely collected NHS datasets and the newly collected PROMs and tissue data will be brought together through the UK Bowel Cancer Intelligence Hub. Descriptive statistics will be used to report the survey results and assess the quality of life outcomes of the participants. The outcomes will be analysed according to stage of disease, treatment type, comorbidity, age, ethnic and sociodemographic group (and other relevant variables). These descriptive analyses will identify potential relationships of interest which can be investigated further. Regression modelling will be used to investigate associations amongst the different types of variables to identify statistically and clinically significant risk factors and predictors of outcomes. In order to be robust, analyses will require appropriate adjustment for case-mix and other confounding factors and may require more complex techniques, such as the modelling of hierarchies within the data (multilevel modelling), post-hoc weighting to overcome response bias and multiple imputation of missing data.

Intervention

The analyses will be disseminated and discussed by regional MDTs at events for each clinical discipline. Agreements of educational needs to improve care will be agreed along with any additional data capture and audit processes that teams agree are required. The process is repeated with ongoing data analyses to establish the improvements in management and outcomes that have occurred (Figure 1). As YCR BCIP progresses, further work will be undertaken looking at other outcomes including, but not limited to, those related to screening, pathology and long-term outcomes.

Public Patient Involvement

Patients and carers were actively engaged through the PROMS Working Group to develop the design and content of the patient questionnaires, the patient information sheet and consent form. At the request of the patients and carers, additional questions were included around the financial impact of cancer and a specific request was made to EORTC to amend the EORTC colorectal module and add specific questions from other EORTC modules to

For peer review only - http://bmjopen.bmj.com/site/about/guidelines.xhtml

understand side effects of cancer and cancer treatments. EORTC granted these specific amendments for this study. The electronic and paper copies of the final draft questionnaires were tested with patients attending a colorectal cancer follow-up clinic at one of the region hospitals. Modification to the layout of the questionnaires were made following the results of the testing. The testing gave an understanding of the length of time it took patients to complete the questionnaires. The PROMS Working Group will remain active throughout the length of the study; the group will be kept apprised of recruitment levels and early results. It is expected that the patients will advise on the analysis and how the results are communicated to the regional clinical teams and wider audiences.

For peer review only

For peer review only - http://bmjopen.bmj.com/site/about/guidelines.xhtml

Discussion

The YCR BCIP study ambitiously aims to improve outcomes for patients with colorectal cancer across the Yorkshire and Humber and region by in-depth analysis of existing and newly captured data, and by actively engaging local MDTs. In its initial stages, this will be done by demonstrating the variation in the demographics, management and outcomes in the region using routine NHS datasets. However, given the limitations of what can be achieved with existing data, the YCR BCIP is collecting additional data to analyse alongside this with the purpose to better understand what is driving the observed variation.

The PROMs data will enrich other study data and allow for an 'in-depth' description of what life is really like for colorectal cancer patients at diagnosis and a year later. At present, the patient voice is not 'measured' routinely as part of clinical practice, neglecting the impact of illness and treatment on the everyday lives of patients. YCR BCIP will change this with the integration of PROMs administration into clinical practice[15]. The PROMs used in this study have been selected with input from experts in colorectal cancer and psychosocial care: patients, clinical nurse specialists and doctors. Although the length of the survey would not be feasible to administer in everyday practice, information on which PROMs provide the most meaningful data will be obtained. In the future risk-stratified follow-up may incorporate not only clinical indicators but also key quality of life indicators to inform best supportive care[38].

Results from the tissue collection and testing could impact on treatment and follow-up decisions for the participating patients and, potentially, their families. For example, the results may indicate that a patient could benefit from a targeted treatment being tested through an open clinical trial if they develop an indication for further treatment e.g. Medical Research Council FOCUS4 [39]. Increased risks of having a hereditary condition may be identified, which not only has implications for the patient but also their family.

For peer review only - http://bmjopen.bmj.com/site/about/guidelines.xhtml

Ethics and dissemination

The study was granted ethical approval (17/WM/0374) by the West Midlands – Solihull Research Ethics Committee in December 2017. The study was approved by the Health Research Authority and granted approval for inclusion in the National Institute for Health Research's portfolio of studies in December 2017 (Project ID 227673).

The YCR BCIP is offering a novel approach to address the variation in colorectal cancer care across a large region of the UK. Engagement of the region's MDTs with their data will lead to a range of educational initiatives, studies and clinical audits that aim to optimise practice across the region. It is planned that the outcomes of these will be presented to the relevant specialty group for review and to develop actions based on findings. Further work from the seven specialty groups involved in the programme: surgery, radiology, pathology, clinical oncology, medical oncology, nursing and anaesthetics, will be reported soon.

Authors' contributions

PQ is the principal investigator.NW, PW and EM are coinvestigators and/or workstream leads. Together these authors conceived and designed the study. HR and JM manage the study. JT provides statistical support. AG provides pathology support. All authors contributed to writing of the manuscript and have approved a final version.

Competing interests

The authors declare that they have no competing interests.

Funding

This work was funded by Yorkshire Cancer Research (Award reference number L394). Prof Quirke holds an NIHR Senior Investigator award. The UK Bowel Cancer Intelligence Hub is supported by Cancer Research UK (C23434/A23706).

Acknowledgements

The YCR BCIP study group includes Philip Quirke, Paul Finan, Penny Wright, Nicholas West, Matthew Seymour, Eva Morris, David Sebag-Montefoire, Daniel Swinson, Damian Tolan, Simon Howell, Peter Brown, John Taylor, Amy Glover, Aidan Hindley, Hannah Rossington,

For peer review only - http://bmjopen.bmj.com/site/about/guidelines.xhtml

Jackie Mara. The YCR BCIP research team acknowledges the support of the National Institute for Health Research Clinical Research Network (NIHR CRN).

For peer review only

For peer review only - http://bmjopen.bmj.com/site/about/guidelines.xhtml

References

1. Allemani C, Matsuda T, Di Carlo V, Harewood R, Matz M, Nikšić M, Bonaventure A, Valkov M, Johnson CJ, Estève J *et al*: **Global surveillance of trends in cancer survival 2000–14 (CONCORD-3): analysis of individual records for 37 513 025 patients diagnosed with one of 18 cancers from 322 population-based registries in 71 countries**. *The Lancet* 2018.
2. Heald RJ, Ryall RD: **Recurrence and survival after total mesorectal excision for rectal cancer**. *Lancet* 1986, **1**(8496):1479-1482.
3. Iversen LH, Green A, Ingeholm P, Osterlind K, Gogenur I: **Improved survival of colorectal cancer in Denmark during 2001-2012 - The efforts of several national initiatives**. *Acta Oncol* 2016, **55 Suppl 2**:10-23.
4. Wibe AM, B.; Norstein, J.; Carlsen, E.; Wiig, J.N.; Heald, R.J.; Langmark, F.; Myrvold, H.E.; Soreide, O.; for the Norwegian Rectal Cancer Group: **A National Strategic Change in Treatment Policy for Rectal Cancer - Implementation of Total Mesorectal Excision as Routine Treatment in Norway. A National Audit**. *Dis Colon Rectum* 2002, **45**(7):857-866.
5. Wibe A, Moller B, Norstein J, Carlsen E, Wiig JN, Heald RJ, Langmark F, Myrvold HE, Soreide O, Norwegian Rectal Cancer G: **A national strategic change in treatment policy for rectal cancer--implementation of total mesorectal excision as routine treatment in Norway. A national audit**. *Dis Colon Rectum* 2002, **45**(7):857-866.
6. Martling AL, Holm T, Rutqvist LE, Moran BJ, Heald RJ, Cedermark B: **Effect of a surgical training programme on outcome of rectal cancer in the County of Stockholm**. *The Lancet* 2000, **356**(9224):93-96.
7. Martling A, Holm T, Rutqvist LE, Johansson H, Moran BJ, Heald RJ, Cedermark B: **Impact of a surgical training programme on rectal cancer outcomes in Stockholm**. *Br J Surg* 2005, **92**(2):225-229.
8. Jessop J, Beagley C, Heald RJ: **The Pelican Cancer Foundation and The English National MDT-TME Development Programme**. *Colorectal Dis* 2006, **8 Suppl 3**:1-2.
9. Moran BJ, Holm T, Brannagan G, Chave H, Quirke P, West N, Brown G, Glynne-Jones R, Sebag-Montefiore D, Cunningham C *et al*: **The English national low rectal cancer development programme: key messages and future perspectives**. *Colorectal Dis* 2014, **16**(3):173-178.
10. Allemani C, Matsuda T, Di Carlo V, Harewood R, Matz M, Niksic M, Bonaventure A, Valkov M, Johnson CJ, Esteve J *et al*: **Global surveillance of trends in cancer survival 2000-14 (CONCORD-3): analysis of individual records for 37 513 025 patients diagnosed with one of 18 cancers from 322 population-based registries in 71 countries**. *Lancet* 2018.
11. **Abstracts of the Association of Coloproctology of Great Britain and Ireland Annual Meeting, 3-5 July 2017, Bournemouth, UK**. *Colorectal Dis* 2017, **19 Suppl 4**:3-66.
12. **UK Bowel Cancer Intelligence Hub** [https://bci.leeds.ac.uk/]
13. **National Cancer Registration and Analysis Service** [http://www.ncr.nhs.uk/]
14. Ashley L, Jones H, Thomas J, Newsham A, Downing A, Morris E, Brown J, Velikova G, Forman D, Wright P: **Integrating patient reported outcomes with clinical cancer registry data: a feasibility study of the electronic Patient-Reported Outcomes From Cancer Survivors (ePOCS) system**. *J Med Internet Res* 2013, **15**(10):e230.

For peer review only - http://bmjopen.bmj.com/site/about/guidelines.xhtml

15. Black N, Burke L, Forrest CB, Sieberer UH, Ahmed S, Valderas JM, Bartlett SJ, Alonso J: **Patient-reported outcomes: pathways to better health, better services, and better societies**. *Qual Life Res* 2016, **25**(5):1103-1112.
16. Ashley L, Jones H, Thomas J, Forman D, Newsham A, Morris E, Johnson O, Velikova G, Wright P: **Integrating cancer survivors' experiences into UK cancer registries: design and development of the ePOCS system (electronic Patient-reported Outcomes from Cancer Survivors)**. *Br J Cancer* 2011, **105 Suppl 1**:S74-81.
17. Foster C, Haviland J, Winter J, Grimmett C, Seymour KC, Batehup L, Calman L, Corner J, Din A, Fenlon D *et al*: **Pre-Surgery Depression and Confidence to Manage Problems Predict Recovery Trajectories of Health and Wellbeing in the First Two Years following Colorectal Cancer: Results from the CREW Cohort Study**. *Plos One* 2016, **11**(5).
18. Ganesh V, Agarwal A, Popovic M, Cella D, McDonald R, Vuong S, Lam H, Rowbottom L, Chan S, Barakat T *et al*: **Comparison of the FACT-C, EORTC QLQ-CR38, and QLQ-CR29 quality of life questionnaires for patients with colorectal cancer: a literature review**. *Supportive Care in Cancer* 2016, **24**(8):3661-3668.
19. Gilbert A, Sebag-Montefiore D, Davidson S, Velikova G: **Use of patient-reported outcomes to measure symptoms and health related quality of life in the clinic**. *Gynecologic Oncology* 2015, **136**(3):429-439.
20. Hadi M, Gibbons E, Fitzpatrick R: **A structured review of patient reported outcome measures for colorectal cancer**. In: *Patient Reported Outcome Measures.* Edited by Patient Reported Outcome Measurement Group UoO. Oxford; 2010.
21. McNair AGK, Whistance RN, Forsythe RO, Rees J, Jones JE, Pullyblank AM, Avery KNL, Brookes ST, Thomas MG, Sylvester PA *et al*: **Synthesis and summary of patient-reported outcome measures to inform the development of a core outcome set in colorectal cancer surgery**. *Colorectal Dis* 2015, **17**(11):O217-O229.
22. Wong CKH, Chen J, Yu CLY, Sham MS, Lam CLK: **Systematic review recommends the European Organization for Research and Treatment of Cancer colorectal cancer-specific module for measuring quality of life in colorectal cancer patients**. *Journal of Clinical Epidemiology* 2015, **68**(3):266-278.
23. McNair AG, Whistance RN, Forsythe RO, Macefield R, Rees J, Pullyblank AM, Avery KN, Brookes ST, Thomas MG, Sylvester PA *et al*: **Core Outcomes for Colorectal Cancer Surgery: A Consensus Study**. *PLoS Med* 2016, **13**(8):e1002071.
24. Aaronson NK, Ahmedzai S, Bergman B, Bullinger M, Cull A, Duez NJ, Filiberti A, Flechtner H, Fleishman SB, de Haes JC *et al*: **The European Organization for Research and Treatment of Cancer QLQ-C30: a quality-of-life instrument for use in international clinical trials in oncology**. *Journal of the National Cancer Institute* 1993, **85**(5):365-376.
25. Greimel E, Nordin A, Lanceley A, Creutzberg CL, van de Poll-Franse LV, Radisic VB, Galalae R, Schmalz C, Barlow E, Jensen PT *et al*: **Psychometric validation of the European Organisation for Research and Treatment of Cancer Quality of Life Questionnaire-Endometrial Cancer Module (EORTC QLQ-EN24)**. *Eur J Cancer* 2011, **47**(2):183-190.
26. Greimel ER, Kuljanic Vlasic K, Waldenstrom AC, Duric VM, Jensen PT, Singer S, Chie W, Nordin A, Bjelic Radisic V, Wydra D *et al*: **The European Organization for Research and Treatment of Cancer (EORTC) Quality-of-Life questionnaire cervical cancer module: EORTC QLQ-CX24**. *Cancer* 2006, **107**(8):1812-1822.

For peer review only - http://bmjopen.bmj.com/site/about/guidelines.xhtml

27. van Andel G, Bottomley A, Fossa SD, Efficace F, Coens C, Guerif S, Kynaston H, Gontero P, Thalmann G, Akdas A *et al*: **An international field study of the EORTC QLQ-PR25: a questionnaire for assessing the health-related quality of life of patients with prostate cancer**. *Eur J Cancer* 2008, **44**(16):2418-2424.
28. Whistance RN, Conroy T, Chie W, Costantini A, Sezer O, Koller M, Johnson CD, Pilkington SA, Arraras J, Ben-Josef E *et al*: **Clinical and psychometric validation of the EORTC QLQ-CR29 questionnaire module to assess health-related quality of life in patients with colorectal cancer**. *Eur J Cancer* 2009, **45**(17):3017-3026.
29. Herdman M, Gudex C, Lloyd A, Janssen M, Kind P, Parkin D, Bonsel G, Badia X: **Development and preliminary testing of the new five-level version of EQ-5D (EQ-5D-5L)**. *Qual Life Res* 2011, **20**(10):1727-1736.
30. Wright EP, Kiely M, Johnston C, Smith AB, Cull A, Selby PJ: **Development and evaluation of an instrument to assess social difficulties in routine oncology practice**. *Qual Life Res* 2005, **14**(2):373-386.
31. Wright P, Smith AB, Keding A, Velikova G: **The Social Difficulties Inventory (SDI): development of subscales and scoring guidance for staff**. *Psychooncology* 2011, **20**(1):36-43.
32. Tennant R, Hiller L, Fishwick R, Platt S, Joseph S, Weich S, Parkinson J, Secker J, Stewart-Brown S: **The Warwick-Edinburgh Mental Well-being Scale (WEMWBS): development and UK validation**. *Health Qual Life Outcomes* 2007, **5**:63.
33. Bartram DJ, Sinclair JM, Baldwin DS: **Further validation of the Warwick-Edinburgh Mental Well-being Scale (WEMWBS) in the UK veterinary profession: Rasch analysis**. *Qual Life Res* 2013, **22**(2):379-391.
34. Lorig KR, Sobel DS, Ritter PL, Laurent D, Hobbs M: **Effect of a self-management program on patients with chronic disease**. *Eff Clin Pract* 2001, **4**(6):256-262.
35. Broadbent E, Petrie KJ, Main J, Weinman J: **The brief illness perception questionnaire**. *J Psychosom Res* 2006, **60**(6):631-637.
36. Marti J, Hall PS, Hamilton P, Hulme CT, Jones H, Velikova G, Ashley L, Wright P: **The economic burden of cancer in the UK: a study of survivors treated with curative intent**. *Psychooncology* 2016, **25**(1):77-83.
37. Spiegelhalter DJ: **Funnel plots for comparing institutional performance**. *Stat Med* 2005, **24**(8):1185-1202.
38. Watson EK, Rose PW, Neal RD, Hulbert-Williams N, Donnelly P, Hubbard G, Elliott J, Campbell C, Weller D, Wilkinson C: **Personalised cancer follow-up: risk stratification, needs assessment or both?** *Br J Cancer* 2012, **106**(1):1-5.
39. Kaplan R, Maughan T, Crook A, Fisher D, Wilson R, Brown L, Parmar M: **Evaluating many treatments and biomarkers in oncology: a new design**. *J Clin Oncol* 2013, **31**(36):4562-4568.

For peer review only - http://bmjopen.bmj.com/site/about/guidelines.xhtml

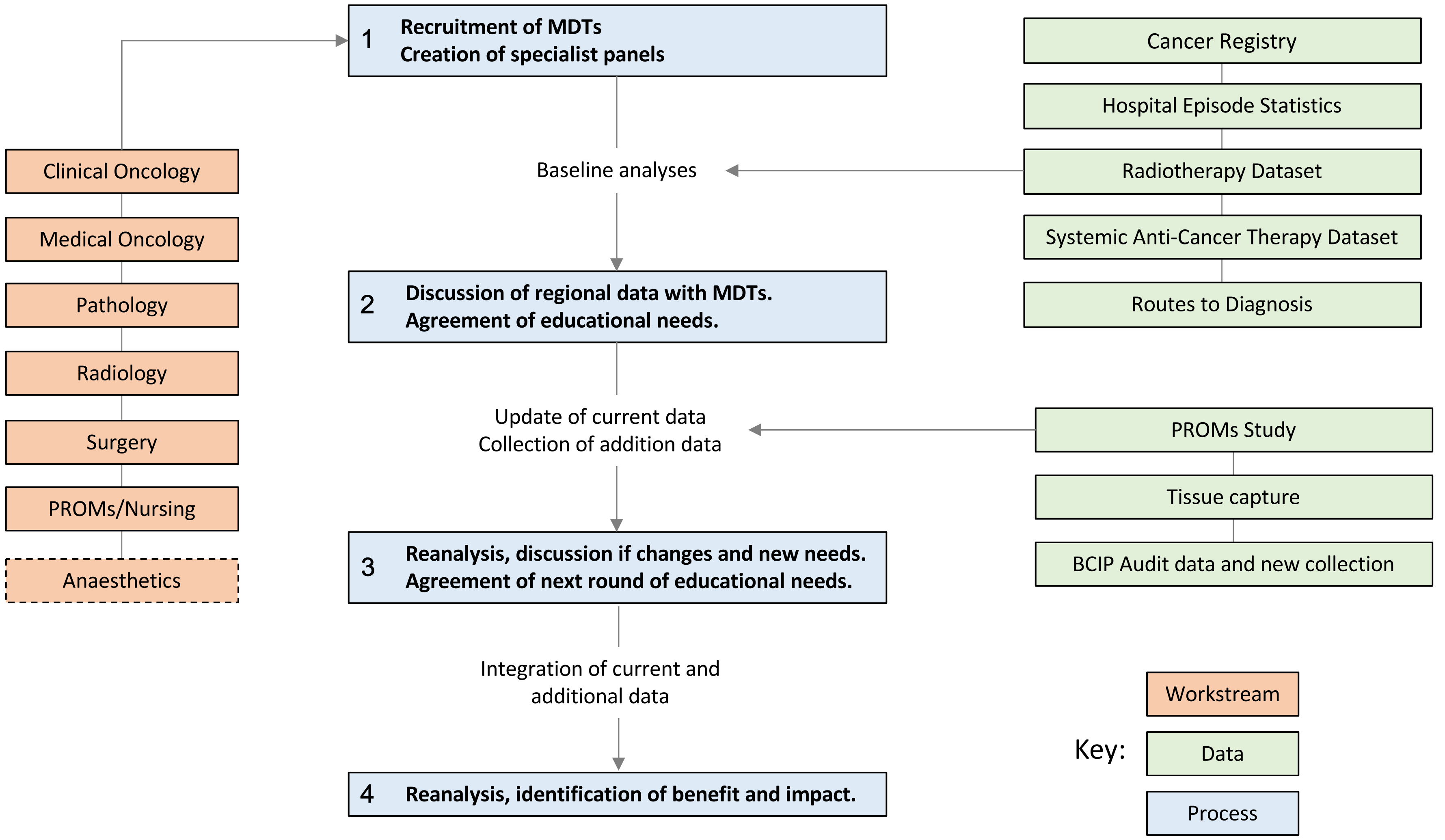
1 Recruitment of MDTs
Creation of specialist panels
Baseline analyses
2 Discussion of regional data with MDTs.
Agreement of educational needs.
Update of current data
Collection of addition data
3 Reanalysis, discussion if changes and new needs.
Agreement of next round of educational needs.
Integration of current and additional data
4 Reanalysis, identification of benefit and impact.
Clinical Oncology
Medical Oncology
Pathology
Radiology
Surgery
PROMs/Nursing
Anaesthetics
Cancer Registry
Hospital Episode Statistics
Radiotherapy Dataset
Systemic Anti-Cancer Therapy Dataset
Routes to Diagnosis
PROMs Study
Tissue capture
BCIP Audit data and new collection
Key:
Workstream
Data
Process

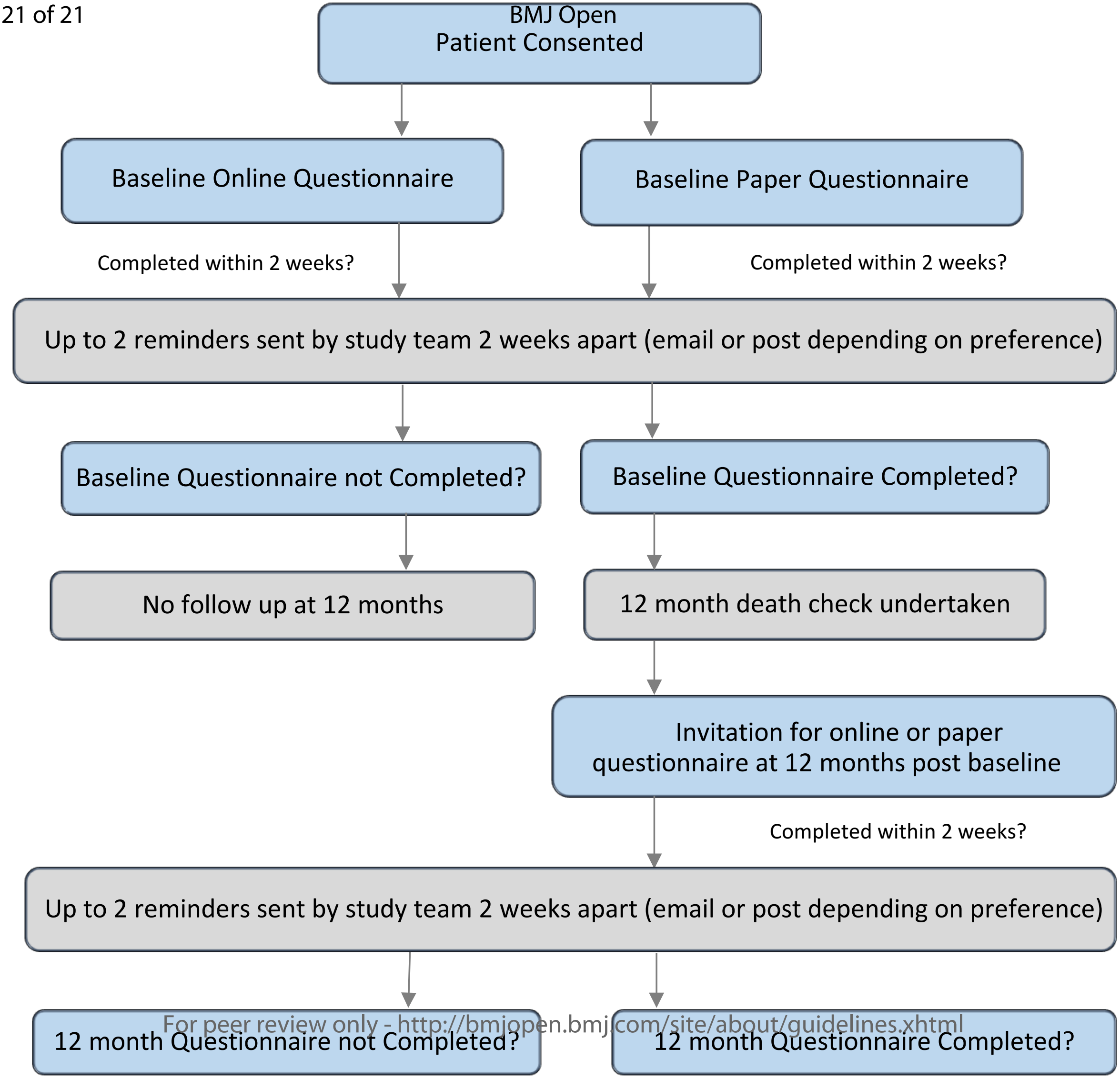

For peer review only - http://bmjopen.bmj.com/site/about/guidelines.xhtml

BMJ Open

The Yorkshire Cancer Research Bowel Cancer Improvement Programme (YCR BCIP): study protocol

Journal:	*BMJ Open*
Manuscript ID	bmjopen-2019-030618.R1
Article Type:	Protocol
Date Submitted by the Author:	23-Jul-2019
Complete List of Authors:	Taylor, John; Leeds Institute of Cancer and Pathology, Section of Epidemiology and Biostatistics, University of Leeds<br>Wright, Penny; Leeds Institute of Cancer and Pathology, Section of Patient Centred Outcome Research, University of Leeds<br>Rossington, Hannah; Leeds Institute of Cancer and Pathology, Section of Epidemiology and Biostatistics, University of Leeds<br>Mara, Jackie; Leeds Institute of Cancer and Pathology, Section of Epidemiology and Biostatistics, University of Leeds<br>Glover, Amy; Leeds Institute of Cancer and Pathology, Section of Pathology and Tumour Biology, University of Leeds<br>West, Nick; Leeds Institute of Cancer and Pathology, Section of Pathology and Tumour Biology, University of Leeds<br>Morris, Eva; Leeds Institute of Cancer and Pathology, Section of Epidemiology and Biostatistics, University of Leeds<br>Quirke, Phillip; Leeds Institute of Cancer and Pathology, Section of Pathology and Tumour Biology, University of Leeds
**Primary Subject Heading**:	Epidemiology
Secondary Subject Heading:	Health policy, Medical education and training, Oncology, Research methods
Keywords:	Quality in health care < HEALTH SERVICES ADMINISTRATION & MANAGEMENT, Gastrointestinal tumours < ONCOLOGY, PUBLIC HEALTH, STATISTICS & RESEARCH METHODS, EPIDEMIOLOGY

SCHOLARONE™
Manuscripts

The Yorkshire Cancer Research Bowel Cancer Improvement Programme (YCR BCIP): study protocol

J.C. Taylor[1*], P. Wright[2], H.L. Rossington[1], J. Mara[1], A. Glover[3], N.P. West[3], E.J.A. Morris[1], P. Quirke[3] on behalf of YCR BCIP Study Group.

1. Cancer Epidemiology Group, Section of Epidemiology and Biostatistics, Leeds Institute of Cancer & Pathology and Leeds Institute for Data Analytics, University of Leeds, Leeds, UK
2. Section of Patient Centred Outcome Research, Leeds Institute of Cancer & Pathology, University of Leeds, Leeds, UK
3. Section of Pathology, Leeds Institute of Medical Research at St. James's, University of Leeds, Leeds, UK

Corresponding author:

J. C. Taylor

Leeds Institute for Data Analytics

Worsley Building

University of Leeds

LS2 9NL, Leeds, UK

j.c.taylor@leeds.ac.uk

For peer review only

For peer review only - http://bmjopen.bmj.com/site/about/guidelines.xhtml

Abstract

Introduction

Although colorectal cancer outcomes in England are improving they remain poorer than many comparable countries. Yorkshire Cancer Research have, therefore, established a Bowel Cancer Improvement Programme (YCR BCIP) to improve colorectal cancer outcomes within Yorkshire and Humber, a region representative of the nation in these terms. It aims to do this by quantifying variation in practice, engaging with the colorectal multidisciplinary teams (MDTs) to understand this and instigating educational interventions to minimise it and improve outcomes.

Methods and analysis

Initially, routine health datasets will be used to quantify variation in the demographics, management and outcomes of patients across the Yorkshire and Humber region and results presented to MDTs. The YCR BCIP is seeking to supplement these existing data with patient reported health-related quality of life information (PROMs) and tissue sample analysis. Specialty groups (surgery, radiology, pathology, clinical oncology, medical oncology, clinical nurse specialists and anaesthetics) have been established to provide oversight and direction for their clinical area within the programme, to review data and analysis and to develop appropriate educational initiatives.

Ethics and dissemination

The YCR BCIP is seeking to address the variation in practice to significantly improve colorectal cancer outcomes across the Yorkshire and Humber region. PROMs and tissue sample collection and analysis will help to capture the information required to fully assess care in the region. Engagement of the region's MDTs with their data will lead to a range of educational initiatives, studies and clinical audits that aim to optimise practice across the region.

For peer review only - http://bmjopen.bmj.com/site/about/guidelines.xhtml

Stengths and limitations of this study

- A novel approach by providing regional colorectal MDTs with data and, with their input, develop educational interventions to minimise any variation seen in order to optimise outcomes.
- Evaluating variation in the management and outcomes of colorectal cancer patients by combining routinely collected clinical datasets and novel information on aspects of care not currently quantifiable through existing datasets.
- The study will facilitate implementation of guidelines such as routine screening for Lynch syndrome and deficient mismatch repair status.

For peer review only

For peer review only - http://bmjopen.bmj.com/site/about/guidelines.xhtml

Introduction

Colorectal cancer is a common disease in the UK with over 41,000 people diagnosed each year and 16,000 people dying from it. Although survival rates have improved over time, they continue to lag behind those attained by many economic neighbours[1] and so developing educational interventions to improve outcomes is a priority.

Some potential explanations for these survival differences include variability in the quality of care and access to services. For example, local recurrence and survival rates in rectal cancer are related to the quality of the surgical resection[2] so improving surgical quality is likely to improve outcomes. Likewise, ensuring gold standard diagnostic, staging and multidisciplinary care across all aspects of the patient pathway have a demonstrable impact on outcomes[3] while screening for Lynch syndrome and deficient mismatch repair will enable more effective managemement of patients.

Other countries have acknowledged these relationships and taken steps to improve standards. For instance, in the early 1990s, Norway recognised there was huge variation in local recurrence rates depending on which surgeon operated[4]. In response, they initiated a training programme to quantify variation and improve the quality of surgery. This strategy was a success, reducing local recurrence and improving overall survival[5]. As a result similar programmes in other countries, including the UK were established with equally positive outcomes including lower postoperative mortality[6] and better survival[3, 7], lower permanent stoma[7] and local recurrence rates[8], better preoperative staging[9], improved selection of patients for non-surgical treatment[9] and the need for less emergency surgery[3].

Whilst these programmes have undoubtedly had a positive impact, variations in management remain and particularly so in the UK. Here, although survival rates have improved, the gap to rates attained in comparable countries[10] have not closed. Better understanding of what is driving these variations will help to target educational interventions to improve care.

Yorkshire Cancer Research is a charity whose aim is to ensure that more people of Yorkshire and Humber avoid, survive and cope with cancer and, by 2025, they intend to have reduced cancer deaths in the region by 2,000 per year. As part of their strategy to achieve this they

For peer review only - http://bmjopen.bmj.com/site/about/guidelines.xhtml

have recently funded the Bowel Cancer Improvement Programme (YCR BCIP). This initiative builds upon the experience gained from several English National programmes [9, 11] and extends the approach of those successful programmes completed in Scandinavia [3, 8, 12]. It centres on the collection and analysis of robust colorectal cancer data to examine practice across the region, ensuring the multidisciplinary teams (MDTs) managing the disease engage with these data and agree areas for improvement. Educational interventions or other strategies can then be implemented to ensure optimal practice is achieved.

The aims of the YCR BCIP study are to:

1. Quantify and report the variation in the demographics, management and outcomes of the region's colorectal cancer patients using
    a. routinely collected clinical datasets,
    b. new information on aspects of care not currently quantifiable through existing datasets (e.g. Patient Reported Outcome Measures (PROMs), the molecular subtyping of the disease and the quality of radiology, pathology and surgery).
2. Where comparative data are available, to determine how outcomes from the Yorkshire and Humber region compare to the rest of England.
3. To provide the Yorkshire and Humber region colorectal MDTs with these data and, with their input, develop educational interventions to minimise any variation seen in order to optimise outcomes
4. Facilitate implementation of guidelines such as routine screening for Lynch syndrome and deficient mismatch repair status.
5. Evaluate improvement in outcomes over the study period.

For peer review only

For peer review only - http://bmjopen.bmj.com/site/about/guidelines.xhtml

Methods and analysis

Setting

The Yorkshire and Humber region of the UK has a population of approximately 5.7 million with around 3,300 colorectal cancer diagnoses a year, amounting to just over 10% of English diagnoses. Patients diagnosed with colorectal cancer in the region are managed by 16 different MDTs. These operate across 14 National Health Service (NHS) Trusts which provide health services for particular geographical areas of the region which exhibit a range of workloads, ranging between approximately 120-390 cases a year (4%-12% of the regional workload). Patients within the geographical areas covered by each Trust exhibit a variable range of demographic features such as socioeconomic deprivation and comorbidity [13].

Programme Overview

YCR BCIP will interrogate routinely collected NHS data and, subject to patient consent, additional data collected specifically for the programme. Baseline performance outcomes for each MDT from across the region will be assessed. The knowledge will be fed back to each MDT and a programme of educational interventions developed based on the performance outcomes. The process will be iterative as outlined in Figure 1. The programme will run from 1st April 2016 until 31 March 2021.

Following a regional meeting of MDT representatives to discuss, plan and agree to programme participation held in June 2016, six specialty groups were initially established. The remit of these groups is to provide oversight and direction for their clinical specialty within the programme, to review data and analyses and to develop appropriate educational initiatives. The six groups focused on the main clinical disciplines involved in the MDT; surgery, radiology, pathology, clinical oncology, medical oncology, and specialist nursing. Group membership was drawn from all of the region's MDTs and an individual established from each to to act as a coordinator between the research team and MDT. The need for a seperate anaesthetics workstream was subsequently identified and introduced.

Study Cohort/Participants

Although broad ranging, it was recognised that the routine colorectal cancer data available is not sufficient to quantify all aspects of care robustly. Whilst routine data provides good information available on 'hard' outcomes such as which operation was undertaken or

For peer review only - http://bmjopen.bmj.com/site/about/guidelines.xhtml

survival time, there is a lack of information on what this really means to the individual concerned and the quality of that survival. This precludes the production of the rich intelligence the MDTs want to fully understand variation in care. Therefore data collated in YCR BCIP will come from three main sources:

Existing population-based datasets

Consisting of routine NHS datasets providing both regional data (around 3,300 cases per year) and comparative national data (around 28,700 cases per year) on patient and tumour characteristics, treatment choices, diagnosis pathways and patient outcomes.

Direct PROMs and tissue collection

Consisting of regional PROMs data collected directly from patients on health-related quality of life (at both the time of diagnosis, before primary treatment if possible and again at 12 months post diagnosis) and molecular testing of tumour and tumour-associated normal mucosal tissue samples.

All MDTs in the region have been invited to participate in this element of the YCR BCIP. Every colorectal cancer patient (ICD10 C18-C20) is eligible if they are considered suitable for treatment, English language literate, aged at least 18 years old and with capacity to give informed consent. Participants will be asked to consent to the study prior to commencement of primary treatment or following an emergency intervention.

Audit and survey data

Consisting of unlinked anonymised data collected from regional MDTs in the form of an audit (specimen photographs or scans for example) or a clinician survey. The exact nature of these shall be identified by each clinical discipline depending on the needs and availability of existing data.

Cohort Idenitification and Patient Recruitment

Existing population-based datasets

All patients diagnosed with a first primary colorectal cancer (ICD10 C18-C20) in England from 1st January 2005 until the end of the study will be obtained from the National Cancer Registration and Analysis Service (NCRAS) [14] and assigned a managing MDT.

Direct PROMs and tissue collection

This research project is adopted by the NIHR Yorkshire and Humber Clinical Research Network and therefore recruitment will be undertaken across the region by network

For peer review only - http://bmjopen.bmj.com/site/about/guidelines.xhtml

research and clinical staff working collaboratively. Recruitment will run over a 30 month period.

Eligible patients will be identified and approached in two ways.

1. Identified via the MDT and informed about the study by consultant letter sent out with their appointment letter for the primary pre-assessment clinic visit . Where possible, patients will be approached about the study at this clinic appointment. Patients missed at this appointment will be contacted at the earliest convenient time point and informed about the study.

2. Identified by their NHS clinical team if they present as an acute admission (for example with a bowel obstruction) and informed about the study by their clinical team following the emergency intervention (e.g. surgery).

Audit and survey data

No specific recruitment will be performed for these data as only anonymised data used as part of routine patient care will be used. Regional MDT clinicians will be invited to complete online surveys.

Consent

Existing population-based datasets

The data is derived from patient-level information collected by the NHS, as part of the care and support of cancer patients. NCRAS has specific legal permission to collect this information without the need to seek consent, however, patients can ask NCRAS to remove their details from the cancer registry at any time.

Direct PROMs and tissue collection

Identified patients will be provided with full study information (written and verbal) by specialist research nurses or Clinical Nurse Specialists who have undergone Good Clinical Practice (GCP training) to assure the rights, safety and wellbeing of research participants are protected (https://www.nihr.ac.uk/our-research-community/clinical-research-staff/learning-and-development/national-directory/good-clinical-practice/). Written consent may be taken at the time of this approach but patients will be given up to a week to think about study participation. Patients who wish to join the study will be asked to read, complete and sign a consent form, including their contact details name, address and/or

For peer review only - http://bmjopen.bmj.com/site/about/guidelines.xhtml

email address. The person taking consent will also record the patient's date of birth, NHS number and gender.

Patients are asked to consent to their clinical team being informed in the event that clinically relevant laboratory results are found. This includes the possibility of hereditary conditions identified through tumour testing e.g. Lynch syndrome or deficient mismatch repair status. These will be confirmed through routine NHS Clinical Genetics testing after counselling following referral from the local clinical teams with no germline testing taking place through the programme.

Audit and survey data

No specific consent will be needed for these data as only anonymised data used as part of routine patient care will be used.

Data Collection

Existing population-based datasets

The YCR BCIP seeks to make maximum use of routine NHS datasets and these are brought together in the UK Bowel Cancer Intelligence Hub[15] where data from the NCRAS are linked to other datasets relevant to colorectal cancer to provide the richest data possible and enable analysis of the full cancer pathway. These include, but are not limited to: Hospital Episode Statistics, Radiotherapy Dataset, Systematic Anti-Cancer Therapy Datatset and Routes to Diagnosis. This data will cover ten years preceding the start of the programme to provide a baseline and be routinely updated to assess changes throughout it.

Direct PROMs and tissue collection

Participants will have the option of completing the PROMs either online (ePROMs) using the University of Leeds secure questionnaire administration system QTool [16], accessed via the study website, (www.YCRBCIP.leeds.ac.uk) or on paper (pPROMs) with a pre-paid return envelope. They will be asked to complete the survey as soon as possible after consenting. This may be completed while attending hospital at the time of consent or later at home. Just prior to the 12 month follow-up questionnaire the patient status will be checked via NCRAS to confirm that the patient is still alive. Patients will then be sent an email/letter 12 months post-diagnosis by the YCR BCIP research team, inviting them to complete PROMs again (on paper or online according to patient preference). At both time points reminder letters or

For peer review only - http://bmjopen.bmj.com/site/about/guidelines.xhtml

emails will be sent at two weeks and followed up two weeks later (if no response) with the survey being resent with the reminder letter. The process is outlined in Figure 2.

The tissue samples to be collected for this study are:

1. excess pre-treatment, diagnostic biopsy tumour tissue
2. excess tissue following surgical resection (tumour and tumour-associated normal mucosal tissue)
3. excess tumour tissue following the biopsy or resection of distant metastases

The tissue samples will be formalin-fixed and paraffin wax-embedded as per the routine histopathology procedures at the patients' hospital. Tissue blocks will be used for any routine clinical diagnostic procedures required by the hospital before being sent to Pathology at the University of Leeds, and will be available to return to the hospital for further clinical testing if required.

Audit and survey data

No additional patient-level data collection beyond that as part of routine patient care will be performed. Clinicians at regional MDTs will complete surverys anonymously online using www.onlinesurveys.ac.uk (formally Bristol Online Survery).

Data Linkage

Whilst intitally the existing population datasets and direct PROMs and tissue collection will be analysed separately, these will subsequently be linked together together through the UK Bowel Cancer Intelligence Hub. This will provide additional patient characterisitcs for analysis of the PROMs and tissue data.

Data analysis

Existing population-based datasets

Baseline assessments of care in the region are to be performed on individuals diagnosed with colorectal cancer (ICD10 C18-C20) in Yorkshire and Humber, enabling comparison between teams in the region and with national data[13]. Initially this includes using descriptive analysis and statistical methods such as regression modelling, survival analysis and funnel plots [17] comparing the following data: demographic characteristics, tumour characteristics, surgery and oncology management and short and long-term outcomes.

For peer review only - http://bmjopen.bmj.com/site/about/guidelines.xhtml

Some analyses will be rerun periodically over the course of the programme to evaluate the impact on outcomes of specific educational interventions. The measures to be analysed and the sources of these can be found in Table 1.

Direct PROMs and tissue collection

Participants will be asked to complete a range of validated generic, cancer-specific and colorectal cancer-specific PROMs in addition to socio-demographic characteristics. Content has been informed by: clinical relevance, opinion of service users, overall length and participant burden[18], reviews of questionnaires measuring quality of life in colorectal cancer patients[19-23] and UK recommendations for the core outcomes set for trials in colorectal cancer surgery[24].

The survey comprises four sections at baseline and five sections at follow-up. The same PROMs are included at both time points. In addition, at the second time point (12-months post diagnosis) participants will be asked to complete questions about the financial costs of cancer. The time taken to complete the survey is estimated is about 30-35 minutes.

Section One: Your overall health and quality of life (both time points)

- European Organisation for Research and Treatment of Cancer (EORTC) Quality of Life Questionnaire QLQ-C30, the colorectal cancer specific module and items from the prostate, endometrial and cervical cancer modules [25-29]
- EuroQol Group EQ-5D-5L [30]

Section Two: Your everyday life and well-being (both time points)

- Social Difficulties Inventory (SDI-21) [31, 32]
- The Short Warwick-Edinburgh Mental Well-being Scale (SWEMWBS) [33, 34]

Section Three: Manging your health (both time points)

- Self-efficacy for Managing Chronic Disease [35]
- The Brief Illness Perceptions Questionnaire (B-IPQ) [36]

Section Four: The financial cost of cancer (Time 2 only)

- A questionnaire developed in-house based on one used in the ePOCS study[37].

Section Four: Questions about you (Time 1) and Section Five: Questions about you (Time 2)

Self-report socio-demographic and clinical details

Descriptive statistics will be used to report the survey results and assess the quality of life outcomes of the participants. Following data linkage, the outcomes will be analysed

For peer review only - http://bmjopen.bmj.com/site/about/guidelines.xhtml

according to stage of disease, treatment type, comorbidity, age, ethnic and sociodemographic group (and other relevant variables). These descriptive analyses will identify potential relationships of interest which can be investigated further. Regression modelling will be used to investigate associations amongst the different types of variables to identify statistically and clinically significant risk factors and predictors of outcomes. In order to be robust, analyses will require appropriate adjustment for case-mix and other confounding factors and may require more complex techniques, such as the modelling of hierarchies within the data (multilevel modelling), post-hoc weighting to overcome response bias and multiple imputation of missing data.

Based on the number of diagnoses per year, there is potential to consent approximately 8,250 patients from sites across the region over a 30 month recruitment period. However due to eligibility restrictions, consent by MDT and by individuals, administrative/practical issues and staggered site recruitment start dates, the participation rate is likely to be less. Using estimates based on previous studies [38, 39] the consent rate is estimated to be around 42% and the an attrition rate of approximately 35% at the 12 months follow-up point, giving a sample size of at least 1200. This will provide MDTs with a comprehensive descriptive profile of their patients in terms of quality of life outcomes; more complex analyses will be undertaken if sufficient numbers are accrued.

Tissue samples removed at surgery or biopsy which are surplus to routine clinical requirements will be utilised by the research team for upfront testing of novel biomarkers. YCR BCIP will test for novel or rare potentially treatable targets via a range of molecular techniques. The immunohistochemical markers of interest include, HER2, PTEN, PD-1 and PD-L1, amphiregulin and epiregulin, and immune system markers CD3 and CD8. The study will also undertake phenotype analysis by using high-resolution scanned images of tumours using novel algorithms to identify improved prognostic and predictive markers of outcome.

Next Generation Sequencing and/or pyrosequencing will be performed on any extracted DNA to identify tumours with specific gene hotspot mutations including, KRAS, NRAS, BRAF, EGFR and PIK3CA. Other molecular markers linked to causation, outcome and response to therapy will also be investigated e.g. bacterial toxin carriage or fusobacterium nucleatum. Analysis of RNA expression levels of receptor ligands such as amphiregulin and epiregulin, and HER2 and HER3 may also be performed.

For peer review only - http://bmjopen.bmj.com/site/about/guidelines.xhtml

Audit and survey data

The analyses of audit and survery data will be dependent clinical specialities involved and the nature of the data collected.

Data Safeguards

Participant recruitment will be undertaken by each Trust involved in the study. Each research network site will allocate a study ID for potential participants. They will use a University of Leeds secure electronic transfer system every two weeks to inform the YCR BCIP research team of all recruitment activity. This will include the consented patients contact details, date of birth and NHS number to allow for follow-up and to ensure tissue blocks are appropriately labelled for tracking and data linkage. All subsequent participant contact will be undertaken by the central YCR BCIP team.

Storage of all hard copy documents will be in locked metal filing cabinets in research offices of the University of Leeds with secure access building controls. Tissue samples will be used and stored in a scure building with restricted access. Electronic data with pseudonomysed (allocated ID number) patient information will be stored in a secure environment. These files will only be accessible to relevant members of the study analysis team. Where temporary storage of sensitive data is required (e.g. contact details for sending out repeat surveys), files will be accessible only to relevant members of the research team and not stored with any linked data. Members of the analysis team will not have access to any identifiable data. Hard copy data will be kept for 5 years following the end of the study (until 2026) for long term follow-up.

Development of Educational **Interventions**

The analyses will be disseminated and discussed by regional MDTs at events for each clinical discipline. Agreements of educational needs to improve care will be agreed along with any additional data capture and audit processes that teams agree are required. The process is repeated with ongoing data analyses to establish the improvements in management and outcomes that have occurred (Figure 1). As YCR BCIP progresses, further work will be undertaken looking at other outcomes including, but not limited to, those related to screening, pathology and long-term outcomes.

Public Patient Involvement

For peer review only - http://bmjopen.bmj.com/site/about/guidelines.xhtml

Patients and carers were actively engaged through the PROMS Working Group to develop the design and content of the patient questionnaires, the patient information sheet and consent form. At the request of the patients and carers, additional questions were included around the financial impact of cancer and a specific request was made to EORTC to amend the EORTC colorectal module and add specific questions from other EORTC modules to understand side effects of cancer and cancer treatments. EORTC granted these specific amendments for this study. The electronic and paper copies of the final draft questionnaires were tested with patients attending a colorectal cancer follow-up clinic at one of the region hospitals. Modification to the layout of the questionnaires were made following the results of the testing. The testing gave an understanding of the length of time it took patients to complete the questionnaires. The PROMS Working Group will remain active throughout the length of the study; the group will be kept apprised of recruitment levels and early results. It is expected that the patients will advise on the analysis and how the results are communicated to the regional clinical teams and wider audiences.

Discussion

The YCR BCIP study ambitiously aims to improve outcomes for patients with colorectal cancer across the Yorkshire and Humber region by in-depth analysis of existing and newly captured data, and by actively engaging local MDTs. In its initial stages, this will be done by demonstrating the variation in the demographics, management and outcomes in the region using routine NHS datasets. However, given the limitations of what can be achieved with existing data, the YCR BCIP is collecting additional data to analyse alongside this with the purpose to better understand what is driving the observed variation.

The PROMs data will enrich other study data and allow for an 'in-depth' description of what life is really like for colorectal cancer patients at diagnosis and a year later. At present, the patient voice is not 'measured' routinely as part of clinical practice, neglecting the impact of illness and treatment on the everyday lives of patients. YCR BCIP will change this with the integration of PROMs administration into clinical practice[39]. The PROMs used in this study have been selected with input from experts in colorectal cancer and psychosocial care: patients, clinical nurse specialists and doctors. Although the length of the survey would not be feasible to administer in everyday practice, information on which PROMs provide the

For peer review only - http://bmjopen.bmj.com/site/about/guidelines.xhtml

most meaningful data will be obtained. In the future risk-stratified follow-up may incorporate not only clinical indicators but also key quality of life indicators to inform best supportive care[40].

Results from the tissue collection and testing could impact on treatment and follow-up decisions for the participating patients and, potentially, their families. For example, the results may indicate that a patient could benefit from a targeted treatment being tested through an open clinical trial if they develop an indication for further treatment e.g. Medical Research Council FOCUS4 [41]. Increased risks of having a hereditary condition may be identified, which not only has implications for the patient but also their family.

Ethics and dissemination

The study was granted ethical approval (17/WM/0374) by the West Midlands – Solihull Research Ethics Committee in December 2017. The study was approved by the Health Research Authority and granted approval for inclusion in the National Institute for Health Research's portfolio of studies in December 2017 (Project ID 227673).

The YCR BCIP is offering a novel approach to address the variation in colorectal cancer care across a large region of the UK. Engagement of the region's MDTs with their data will lead to a range of educational initiatives, studies and clinical audits that aim to optimise practice across the region. It is planned that the outcomes of these will be presented to the relevant specialty group for review and to develop actions based on findings. Further work from the seven specialty groups involved in the programme: surgery, radiology, pathology, clinical oncology, medical oncology, nursing and anaesthetics, will be reported soon.

Authors' contributions

PQ is the principal investigator.NW, PW and EM are coinvestigators and/or workstream leads. Together these authors conceived and designed the study. HR and JM manage the study. JT provides statistical support. AG provides pathology support. All authors contributed to writing of the manuscript and have approved a final version.

Competing interests

The authors declare that they have no competing interests.

For peer review only - http://bmjopen.bmj.com/site/about/guidelines.xhtml

Funding

This work was funded by Yorkshire Cancer Research (Award reference number L394). Prof Quirke holds an NIHR Senior Investigator award. The UK Bowel Cancer Intelligence Hub is supported by Cancer Research UK (C23434/A23706).

Acknowledgements

The YCR BCIP study group includes Philip Quirke, Paul Finan, Penny Wright, Nicholas West, Matthew Seymour, Eva Morris, David Sebag-Montefoire, Daniel Swinson, Damian Tolan, Simon Howell, Peter Brown, John Taylor, Amy Glover, Aidan Hindley, Hannah Rossington, Jackie Mara. The YCR BCIP research team acknowledges the support of the National Institute for Health Research Clinical Research Network (NIHR CRN).

References

1. Allemani C, Matsuda T, Di Carlo V, Harewood R, Matz M, Nikšić M, Bonaventure A, Valkov M, Johnson CJ, Estève J *et al*: **Global surveillance of trends in cancer survival 2000–14 (CONCORD-3): analysis of individual records for 37 513 025 patients diagnosed with one of 18 cancers from 322 population-based registries in 71 countries**. *The Lancet* 2018.
2. Heald RJ, Ryall RD: **Recurrence and survival after total mesorectal excision for rectal cancer**. *Lancet* 1986, **1**(8496):1479-1482.
3. Iversen LH, Green A, Ingeholm P, Osterlind K, Gogenur I: **Improved survival of colorectal cancer in Denmark during 2001-2012 - The efforts of several national initiatives**. *Acta Oncol* 2016, **55 Suppl 2**:10-23.
4. Holm T, Johansson H, Cedermark B, Ekelund G, Rutqvist LE: **Influence of hospital- and surgeon-related factors on outcome after treatment of rectal cancer with or without preoperative radiotherapy**. *Br J Surg* 1997, **84**(5):657-663.
5. Wibe AM, B.; Norstein, J.; Carlsen, E.; Wiig, J.N.; Heald, R.J.; Langmark, F.; Myrvold, H.E.; Soreide, O.; for the Norwegian Rectal Cancer Group: **A National Strategic Change in Treatment Policy for Rectal Cancer - Implementation of Total Mesorectal Excision as Routine Treatment in Norway. A National Audit**. *Dis Colon Rectum* 2002, **45**(7):857-866.
6. Iversen LH, Ingeholm P, Gogenur I, Laurberg S: **Major reduction in 30-day mortality after elective colorectal cancer surgery: a nationwide population-based study in Denmark 2001-2011**. *Ann Surg Oncol* 2014, **21**(7):2267-2273.
7. Martling A, Holm T, Rutqvist LE, Johansson H, Moran BJ, Heald RJ, Cedermark B: **Impact of a surgical training programme on rectal cancer outcomes in Stockholm**. *Br J Surg* 2005, **92**(2):225-229.
8. Martling AL, Holm T, Rutqvist LE, Moran BJ, Heald RJ, Cedermark B: **Effect of a surgical training programme on outcome of rectal cancer in the County of Stockholm**. *The Lancet* 2000, **356**(9224):93-96.
9. Moran BJ, Holm T, Brannagan G, Chave H, Quirke P, West N, Brown G, Glynne-Jones R, Sebag-Montefiore D, Cunningham C *et al*: **The English national low rectal cancer**

For peer review only - http://bmjopen.bmj.com/site/about/guidelines.xhtml

development programme: key messages and future perspectives. *Colorectal Dis* 2014, **16**(3):173-178.
10. Allemani C, Matsuda T, Di Carlo V, Harewood R, Matz M, Niksic M, Bonaventure A, Valkov M, Johnson CJ, Esteve J *et al*: **Global surveillance of trends in cancer survival 2000-14 (CONCORD-3): analysis of individual records for 37 513 025 patients diagnosed with one of 18 cancers from 322 population-based registries in 71 countries**. *Lancet* 2018.
11. Jessop J, Beagley C, Heald RJ: **The Pelican Cancer Foundation and The English National MDT-TME Development Programme**. *Colorectal Dis* 2006, **8 Suppl 3**:1-2.
12. Wibe A, Moller B, Norstein J, Carlsen E, Wiig JN, Heald RJ, Langmark F, Myrvold HE, Soreide O, Norwegian Rectal Cancer G: **A national strategic change in treatment policy for rectal cancer--implementation of total mesorectal excision as routine treatment in Norway. A national audit**. *Dis Colon Rectum* 2002, **45**(7):857-866.
13. **Abstracts of the Association of Coloproctology of Great Britain and Ireland Annual Meeting, 3-5 July 2017, Bournemouth, UK**. *Colorectal Dis* 2017, **19 Suppl 4**:3-66.
14. **National Cancer Registration and Analysis Service** [http://www.ncr.nhs.uk/]
15. **UK Bowel Cancer Intelligence Hub** [https://bci.leeds.ac.uk/]
16. Ashley L, Jones H, Thomas J, Forman D, Newsham A, Morris E, Johnson O, Velikova G, Wright P: **Integrating cancer survivors' experiences into UK cancer registries: design and development of the ePOCS system (electronic Patient-reported Outcomes from Cancer Survivors)**. *Br J Cancer* 2011, **105 Suppl 1**:S74-81.
17. Spiegelhalter DJ: **Funnel plots for comparing institutional performance**. *Stat Med* 2005, **24**(8):1185-1202.
18. Foster C, Haviland J, Winter J, Grimmett C, Seymour KC, Batehup L, Calman L, Corner J, Din A, Fenlon D *et al*: **Pre-Surgery Depression and Confidence to Manage Problems Predict Recovery Trajectories of Health and Wellbeing in the First Two Years following Colorectal Cancer: Results from the CREW Cohort Study**. *Plos One* 2016, **11**(5).
19. Ganesh V, Agarwal A, Popovic M, Cella D, McDonald R, Vuong S, Lam H, Rowbottom L, Chan S, Barakat T *et al*: **Comparison of the FACT-C, EORTC QLQ-CR38, and QLQ-CR29 quality of life questionnaires for patients with colorectal cancer: a literature review**. *Supportive Care in Cancer* 2016, **24**(8):3661-3668.
20. Gilbert A, Sebag-Montefiore D, Davidson S, Velikova G: **Use of patient-reported outcomes to measure symptoms and health related quality of life in the clinic**. *Gynecologic Oncology* 2015, **136**(3):429-439.
21. Hadi M, Gibbons E, Fitzpatrick R: **A structured review of patient reported outcome measures for colorectal cancer**. In: *Patient Reported Outcome Measures.* Edited by Patient Reported Outcome Measurement Group UoO. Oxford; 2010.
22. McNair AGK, Whistance RN, Forsythe RO, Rees J, Jones JE, Pullyblank AM, Avery KNL, Brookes ST, Thomas MG, Sylvester PA *et al*: **Synthesis and summary of patient-reported outcome measures to inform the development of a core outcome set in colorectal cancer surgery**. *Colorectal Dis* 2015, **17**(11):O217-O229.
23. Wong CKH, Chen J, Yu CLY, Sham MS, Lam CLK: **Systematic review recommends the European Organization for Research and Treatment of Cancer colorectal cancer-specific module for measuring quality of life in colorectal cancer patients**. *Journal of Clinical Epidemiology* 2015, **68**(3):266-278.

For peer review only - http://bmjopen.bmj.com/site/about/guidelines.xhtml

24. McNair AG, Whistance RN, Forsythe RO, Macefield R, Rees J, Pullyblank AM, Avery KN, Brookes ST, Thomas MG, Sylvester PA *et al*: **Core Outcomes for Colorectal Cancer Surgery: A Consensus Study**. *PLoS Med* 2016, **13**(8):e1002071.
25. Aaronson NK, Ahmedzai S, Bergman B, Bullinger M, Cull A, Duez NJ, Filiberti A, Flechtner H, Fleishman SB, de Haes JC *et al*: **The European Organization for Research and Treatment of Cancer QLQ-C30: a quality-of-life instrument for use in international clinical trials in oncology**. *Journal of the National Cancer Institute* 1993, **85**(5):365-376.
26. Greimel E, Nordin A, Lanceley A, Creutzberg CL, van de Poll-Franse LV, Radisic VB, Galalae R, Schmalz C, Barlow E, Jensen PT *et al*: **Psychometric validation of the European Organisation for Research and Treatment of Cancer Quality of Life Questionnaire-Endometrial Cancer Module (EORTC QLQ-EN24)**. *Eur J Cancer* 2011, **47**(2):183-190.
27. Greimel ER, Kuljanic Vlasic K, Waldenstrom AC, Duric VM, Jensen PT, Singer S, Chie W, Nordin A, Bjelic Radisic V, Wydra D *et al*: **The European Organization for Research and Treatment of Cancer (EORTC) Quality-of-Life questionnaire cervical cancer module: EORTC QLQ-CX24**. *Cancer* 2006, **107**(8):1812-1822.
28. van Andel G, Bottomley A, Fossa SD, Efficace F, Coens C, Guerif S, Kynaston H, Gontero P, Thalmann G, Akdas A *et al*: **An international field study of the EORTC QLQ-PR25: a questionnaire for assessing the health-related quality of life of patients with prostate cancer**. *Eur J Cancer* 2008, **44**(16):2418-2424.
29. Whistance RN, Conroy T, Chie W, Costantini A, Sezer O, Koller M, Johnson CD, Pilkington SA, Arraras J, Ben-Josef E *et al*: **Clinical and psychometric validation of the EORTC QLQ-CR29 questionnaire module to assess health-related quality of life in patients with colorectal cancer**. *Eur J Cancer* 2009, **45**(17):3017-3026.
30. Herdman M, Gudex C, Lloyd A, Janssen M, Kind P, Parkin D, Bonsel G, Badia X: **Development and preliminary testing of the new five-level version of EQ-5D (EQ-5D-5L)**. *Qual Life Res* 2011, **20**(10):1727-1736.
31. Wright EP, Kiely M, Johnston C, Smith AB, Cull A, Selby PJ: **Development and evaluation of an instrument to assess social difficulties in routine oncology practice**. *Qual Life Res* 2005, **14**(2):373-386.
32. Wright P, Smith AB, Keding A, Velikova G: **The Social Difficulties Inventory (SDI): development of subscales and scoring guidance for staff**. *Psychooncology* 2011, **20**(1):36-43.
33. Tennant R, Hiller L, Fishwick R, Platt S, Joseph S, Weich S, Parkinson J, Secker J, Stewart-Brown S: **The Warwick-Edinburgh Mental Well-being Scale (WEMWBS): development and UK validation**. *Health Qual Life Outcomes* 2007, **5**:63.
34. Bartram DJ, Sinclair JM, Baldwin DS: **Further validation of the Warwick-Edinburgh Mental Well-being Scale (WEMWBS) in the UK veterinary profession: Rasch analysis**. *Qual Life Res* 2013, **22**(2):379-391.
35. Lorig KR, Sobel DS, Ritter PL, Laurent D, Hobbs M: **Effect of a self-management program on patients with chronic disease**. *Eff Clin Pract* 2001, **4**(6):256-262.
36. Broadbent E, Petrie KJ, Main J, Weinman J: **The brief illness perception questionnaire**. *J Psychosom Res* 2006, **60**(6):631-637.
37. Marti J, Hall PS, Hamilton P, Hulme CT, Jones H, Velikova G, Ashley L, Wright P: **The economic burden of cancer in the UK: a study of survivors treated with curative intent**. *Psychooncology* 2016, **25**(1):77-83.

For peer review only - http://bmjopen.bmj.com/site/about/guidelines.xhtml

38. Ashley L, Jones H, Thomas J, Newsham A, Downing A, Morris E, Brown J, Velikova G, Forman D, Wright P: **Integrating patient reported outcomes with clinical cancer registry data: a feasibility study of the electronic Patient-Reported Outcomes From Cancer Survivors (ePOCS) system**. *J Med Internet Res* 2013, **15**(10):e230.
39. Black N, Burke L, Forrest CB, Sieberer UH, Ahmed S, Valderas JM, Bartlett SJ, Alonso J: **Patient-reported outcomes: pathways to better health, better services, and better societies**. *Qual Life Res* 2016, **25**(5):1103-1112.
40. Watson EK, Rose PW, Neal RD, Hulbert-Williams N, Donnelly P, Hubbard G, Elliott J, Campbell C, Weller D, Wilkinson C: **Personalised cancer follow-up: risk stratification, needs assessment or both?** *Br J Cancer* 2012, **106**(1):1-5.
41. Kaplan R, Maughan T, Crook A, Fisher D, Wilson R, Brown L, Parmar M: **Evaluating many treatments and biomarkers in oncology: a new design**. *J Clin Oncol* 2013, **31**(36):4562-4568.

Figure Legends

Figure 1: Yorkshire Cancer Research Bowel Cancer Improvement Programme (YCRBCIP) process and study design.

Figure 2: Process for the Patient Reported Outcomes Measures (PROMs) study.

For peer review only

For peer review only - http://bmjopen.bmj.com/site/about/guidelines.xhtml

	Data Source		
	**Existing population-based datasets**	**Direct PROMs and tissue collection**	**Audit and survey data**
**Patient and Tumour Characteristics**			
Age & sex	Yes (NCRAS)	Yes	
Ethnicity	Yes (NCRAS)	Yes	
Height & Weight		Yes	
Comorbidity	Yes (NCRAS)	Yes	
Socio-economic status	Yes (NCRAS)	Yes	
Stage & site	Yes (NCRAS)		
Method of admission	Yes (RtD)		
**Treatment Variation**			
Surgical resection rate	Yes (HES)		
Quality of surgery			Yes
Abdominoperineal excision rate	Yes (HES)		
Use of adjuvant & palliative chemotherapy	Yes (SACT)		Yes
Use of neoadjuvant radiotherapy	Yes (RTDS)		
Use of laparoscopic surgery	Yes (HES)		
Emergency care procedures	Yes (HES)		Yes
Practice of anaesthetics			Yes
Quality of MRI reporting			Yes
Quality of CT imaging			Yes
Liver metastases resection rate	Yes (HES)		
Nodal yields and retrieval methods	Yes (NCRAS)		Yes
**Outcomes**			
30-day Postoperative mortality	Yes (NCRAS)		
1 to 5 year overall and net survival	Yes (NCRAS)		
18-month postoperative stoma rate	Yes (HES)		
Postoperative hospital stay	Yes (HES)		
Emergency readmission rates	Yes (HES)		
Overall health and quality of life		Yes	
Everyday life and well-being		Yes	
Self-efficacy for Managing Chronic Disease		Yes	
Financial cost of cancer		Yes	
Urinary function and faecal incontinence		Yes	
Sexual functioning		Yes	
Lower anterior resection syndrome		Yes	
Molecular subtyping		Yes	

Abbreviations: Magnetic resonance imaging (MRI), Computerized Tomography (CT), National Cancer Registration and Analysis Service (NCRAS), Routes to Diagnosis (RtD), Hospital Episode Statistics (HES), Systemic Anti-Cancer Therapy Dataset (SACT), Radiotherapy Dataset (RTDS).

Table 1. Measures of patient characteristics, treatment measures and outcomes to analysed.

For peer review only

For peer review only - http://bmjopen.bmj.com/site/about/guidelines.xhtml

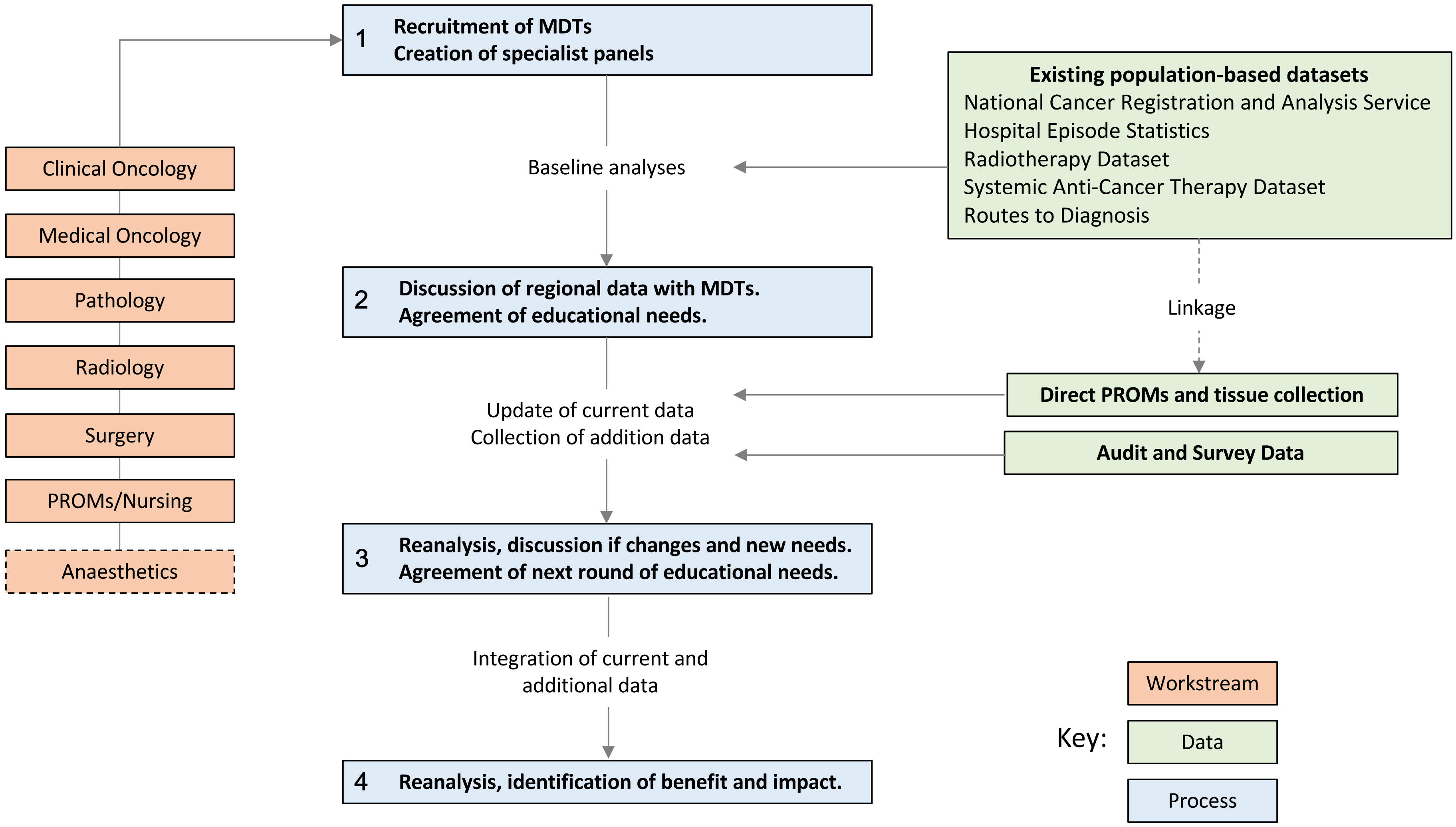
1 Recruitment of MDTs
Creation of specialist panels
Existing population-based datasets
National Cancer Registration and Analysis Service
Hospital Episode Statistics
Radiotherapy Dataset
Systemic Anti-Cancer Therapy Dataset
Routes to Diagnosis
Clinical Oncology
Baseline analyses
Medical Oncology
Pathology
2 Discussion of regional data with MDTs.
Agreement of educational needs.
Linkage
Radiology
Direct PROMs and tissue collection
Update of current data
Collection of addition data
Surgery
Audit and Survey Data
PROMs/Nursing
Anaesthetics
3 Reanalysis, discussion if changes and new needs.
Agreement of next round of educational needs.
Integration of current and
additional data
Workstream
Key:
Data
4 Reanalysis, identification of benefit and impact.
Process

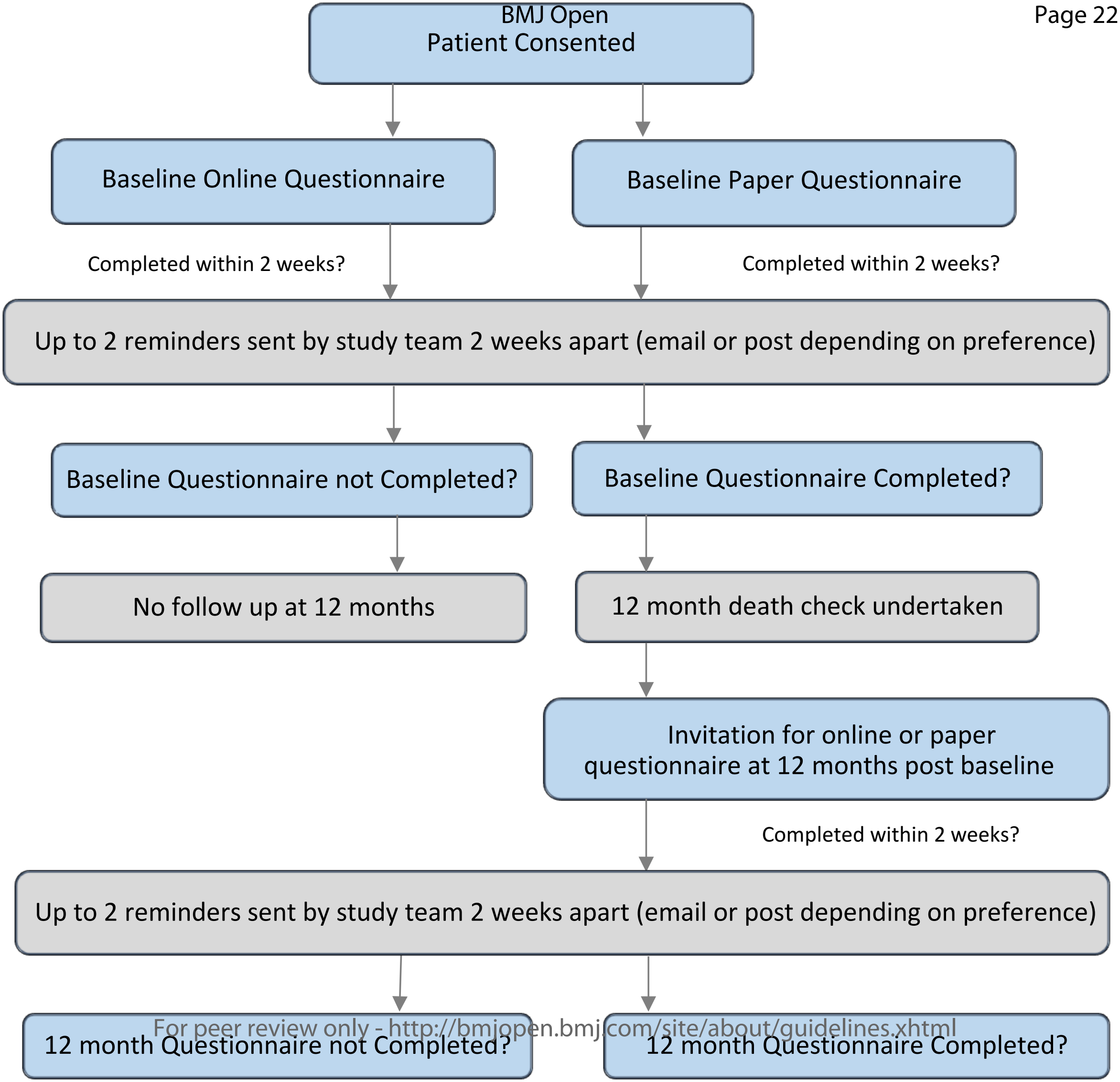

For peer review only - http://bmjopen.bmj.com/site/about/guidelines.xhtml

BMJ Open

A regional intensive multidisciplinary team intervention programme to improve colorectal cancer outcomes: study protocol for the Yorkshire Cancer Research Bowel Cancer Improvement Programme (YCR BCIP)

Journal:	*BMJ Open*
Manuscript ID	bmjopen-2019-030618.R2
Article Type:	Protocol
Date Submitted by the Author:	05-Sep-2019
Complete List of Authors:	Taylor, John; Leeds Institute of Cancer and Pathology, Section of Epidemiology and Biostatistics, University of Leeds<br>Wright, Penny; Leeds Institute of Cancer and Pathology, Section of Patient Centred Outcome Research, University of Leeds<br>Rossington, Hannah; Leeds Institute of Cancer and Pathology, Section of Epidemiology and Biostatistics, University of Leeds<br>Mara, Jackie; Leeds Institute of Cancer and Pathology, Section of Epidemiology and Biostatistics, University of Leeds<br>Glover, Amy; Leeds Institute of Cancer and Pathology, Section of Pathology and Tumour Biology, University of Leeds<br>West, Nick; Leeds Institute of Cancer and Pathology, Section of Pathology and Tumour Biology, University of Leeds<br>Morris, Eva; Leeds Institute of Cancer and Pathology, Section of Epidemiology and Biostatistics, University of Leeds<br>Quirke, Phillip; Leeds Institute of Cancer and Pathology, Section of Pathology and Tumour Biology, University of Leeds
**Primary Subject Heading**:	Epidemiology
Secondary Subject Heading:	Health policy, Medical education and training, Oncology, Research methods
Keywords:	Quality in health care < HEALTH SERVICES ADMINISTRATION & MANAGEMENT, Gastrointestinal tumours < ONCOLOGY, PUBLIC HEALTH, STATISTICS & RESEARCH METHODS, EPIDEMIOLOGY

SCHOLARONE™
Manuscripts

A regional intensive multidisciplinary team intervention programme to improve colorectal cancer outcomes: study protocol for the Yorkshire Cancer Research Bowel Cancer Improvement Programme (YCR BCIP)

J.C. Taylor[1*], P. Wright[2], H.L. Rossington[1], J. Mara[1], A. Glover[3], N.P. West[3], E.J.A. Morris[1], P. Quirke[3] on behalf of YCR BCIP Study Group.

1. Cancer Epidemiology Group, Section of Epidemiology and Biostatistics, Leeds Institute of Cancer & Pathology and Leeds Institute for Data Analytics, University of Leeds, Leeds, UK
2. Section of Patient Centred Outcome Research, Leeds Institute of Cancer & Pathology, University of Leeds, Leeds, UK
3. Section of Pathology, Leeds Institute of Medical Research at St. James's, University of Leeds, Leeds, UK

Corresponding author:

J. C. Taylor

Leeds Institute for Data Analytics

Worsley Building

University of Leeds

LS2 9NL, Leeds, UK

j.c.taylor@leeds.ac.uk

For peer review only

For peer review only - http://bmjopen.bmj.com/site/about/guidelines.xhtml

Abstract

Introduction

Although colorectal cancer outcomes in England are improving they remain poorer than many comparable countries. Yorkshire Cancer Research have, therefore, established a Bowel Cancer Improvement Programme (YCR BCIP) to improve colorectal cancer outcomes within Yorkshire and Humber, a region representative of the nation. It aims to do this by quantifying variation in practice, engaging with the colorectal multidisciplinary teams (MDTs) to understand this and instigating educational interventions to minimise it and improve outcomes.

Methods and analysis

Initially, routine health datasets will be used to quantify variation in the demographics, management and outcomes of patients across the Yorkshire and Humber region and results presented to MDTs. The YCR BCIP is seeking to supplement these existing data with patient reported health-related quality of life information (PROMs) and tissue sample analysis. Specialty groups (surgery, radiology, pathology, clinical oncology, medical oncology, clinical nurse specialists and anaesthetics) have been established to provide oversight and direction for their clinical area within the programme, to review data and analysis and to develop appropriate educational initiatives.

Ethics and dissemination

The YCR BCIP is seeking to address the variation in practice to significantly improve colorectal cancer outcomes across the Yorkshire and Humber region. PROMs and tissue sample collection and analysis will help to capture the information required to fully assess care in the region. Engagement of the region's MDTs with their data will lead to a range of educational initiatives, studies and clinical audits that aim to optimise practice across the region.

For peer review only - http://bmjopen.bmj.com/site/about/guidelines.xhtml

Stengths and limitations of this study

- This study uses a novel approach by providing regional colorectal cancer MDTs with data and, with their input, develop educational interventions to minimise any variation seen in order to optimise outcomes.
- The strength of this study includes evaluating variation in the management and outcomes of colorectal cancer patients by combining routinely collected clinical datasets and novel information on aspects of care not currently quantifiable through existing datasets.
- The study will facilitate implementation of guidelines such as routine screening for Lynch syndrome and deficient mismatch repair status.
- The potential of the study is limited by the extent of engagement from regional colorectal cancer MDTs.

For peer review only

Introduction

Colorectal cancer is a common disease in the UK with over 41,000 people diagnosed each year and 16,000 people dying from it. Although survival rates have improved over time, they continue to lag behind those attained by many economic neighbours[1] and so developing educational interventions to improve outcomes is a priority.

Some potential explanations for these survival differences include quality of care and access to services. For example, local recurrence and survival rates in rectal cancer are related to the quality of the surgical resection[2] so improving surgical quality is likely to improve outcomes. Likewise, ensuring gold standard diagnostic, staging and multidisciplinary care across all aspects of the patient pathway have a demonstrable impact on outcomes[3] while screening for Lynch syndrome and deficient mismatch repair will enable more effective managemement of patients.

Other countries have acknowledged these relationships and taken steps to improve standards. For instance, in the early 1990s, Norway recognised there was huge variation in local recurrence rates depending on which surgeon operated[4]. In response, they initiated a training programme to quantify variation and improve the quality of surgery. This strategy was a success, reducing local recurrence and improving overall survival[5]. As a result similar programmes in other countries, including the UK were established with equally positive outcomes including lower postoperative mortality[6] and better survival[3 7], lower permanent stoma[7] and local recurrence rates[8], better preoperative staging[9], improved selection of patients for non-surgical treatment[9] and the need for less emergency surgery[3].

Whilst these programmes have undoubtedly had a positive impact, variations in management remain and particularly so in the UK[10 11]. Although survival rates in the UK have improved, the gap to rates attained in comparable countries[12] have not closed. Better understanding of what is driving these variations will help to target educational interventions to improve care.

Yorkshire Cancer Research is a charity whose aim is to ensure that more people of Yorkshire and Humber “avoid, survive and cope” with cancer and, by 2025, they intend to have reduced cancer deaths in the region by 2,000 per year[13]. As part of their strategy to achieve this they have recently funded the Bowel Cancer Improvement Programme (YCR BCIP). This

For peer review only - http://bmjopen.bmj.com/site/about/guidelines.xhtml

initiative builds upon the experience gained from English National programmes [9 14] and extends the approach of those successful programmes completed in Scandinavia [3 8 15]. It centres on the collection and analysis of robust colorectal cancer data to examine practice across the region, ensuring the multidisciplinary teams (MDTs) managing the disease engage with these data and agree on areas for improvement. Educational interventions or other strategies can then be developed and implemented to ensure optimal practice is achieved.

This protocol paper gives an overview the YCR BCIP study, which has the following aims:

1. Quantify and report the variation in the demographics, management and outcomes of the region's colorectal cancer patients using
    a. routinely collected clinical datasets,
    b. new information on aspects of care not currently quantifiable through existing datasets (e.g. Patient Reported Outcome Measures (PROMs), the molecular subtyping of the disease and the quality of radiology, pathology and surgery).
2. Determine how outcomes from the Yorkshire and Humber region compare to the rest of England.
3. Provide the Yorkshire and Humber region colorectal MDTs with these data and, with their input, develop educational interventions to minimise any variation seen in order to optimise outcomes
4. Facilitate implementation of guidelines such as routine screening for Lynch syndrome and deficient mismatch repair status.
5. Evaluate improvement in outcomes over the study period.

For peer review only - http://bmjopen.bmj.com/site/about/guidelines.xhtml

Methods and analysis

Setting

The Yorkshire and Humber region of the UK has a population of approximately 5.7 million with around 3,300 colorectal cancer diagnoses a year, amounting to just over 10% of English diagnoses. Patients diagnosed with colorectal cancer in the region are managed by 16 different MDTs. These operate across 14 National Health Service (NHS) Trusts which provide health services for particular geographical areas of the region which exhibit a range of workloads, ranging between approximately 120-390 cases a year (4%-12% of the regional workload). Patients within the geographical areas covered by each Trust exhibit a variable range of demographic features such as socioeconomic deprivation and comorbidity [16].

Programme Overview

YCR BCIP will examine routinely collected NHS data and, subject to patient consent, additional data collected specifically for the programme. Baseline performance outcomes for each MDT from across the region will be assessed. The knowledge will be fed back to each MDT and a programme of educational interventions developed based on the performance outcomes. The process will be iterative as outlined in Figure 1. The programme commenced on 1st April 2016 and will run until 31 March 2021.

Following a regional meeting of MDT representatives to discuss, plan and agree to programme participation held in June 2016, six specialty groups were initially established. The remit of these groups is to provide oversight and direction for their clinical specialty within the programme, to review data and analyses and to develop appropriate educational initiatives. The six groups focused on the main clinical disciplines involved in the MDT; surgery, radiology, pathology, clinical oncology, medical oncology, and specialist nursing. Group membership was drawn from all of the region's MDTs and an individual identified from each to to act as a coordinator between the research team and MDT. The need for a seperate anaesthetics workstream was subsequently identified and introduced. A lead clinician was also identified for each discipline, having the responsibility to coordinate educational events, gather opinion on best practice, formulate consensus views and drive agreed initiatives into routine clinical care across the region.

For peer review only - http://bmjopen.bmj.com/site/about/guidelines.xhtml

Data Sources

Although broad ranging, it was recognised that the routine colorectal cancer data available is not sufficient to quantify all aspects of care robustly. Whilst routine data provides good information available on 'hard' outcomes such as which operation was undertaken or survival time, there is a lack of information on what this really means to the individual concerned and the quality of that survival. This precludes the production of the rich intelligence the MDTs want to fully understand variation in care. Therefore data collated in YCR BCIP will come from three main sources:

Existing population-based datasets

Consisting of routine NHS datasets providing both regional data (around 3,300 cases per year) and comparative national data (around 28,700 cases per year) on patient and tumour characteristics, treatment choices, diagnosis pathways and patient outcomes.

Direct PROMs and tissue collection

Consisting of regional PROMs data collected directly from patients on health-related quality of life (at both the time of diagnosis, before primary treatment if possible and again at 12 months post diagnosis) and molecular testing of tumour and tumour-associated normal mucosal tissue samples.

All MDTs in the region have been invited to participate in this element of the YCR BCIP. Every colorectal cancer patient (ICD10 C18-C20) is eligible if they are considered suitable for treatment, English language literate, aged at least 18 years old and with capacity to give informed consent. Participants will be asked to consent to the study prior to commencement of primary treatment or following an emergency intervention.

Audit and survey data

Consisting of unlinked anonymised data collected from clinicians at regional MDTs in the form of an audit (surgical specimen photographs or scans for example) or a clinician survey. The exact nature of these shall be identified by each clinical discipline depending on the needs and availability of existing data.

Data collection

Existing population-based datasets

The YCR BCIP seeks to make maximum use of routine NHS datasets and these are brought together in the UK Bowel Cancer Intelligence Hub[17] where data from the NCRAS are linked

For peer review only - http://bmjopen.bmj.com/site/about/guidelines.xhtml

to other datasets relevant to colorectal cancer to provide the richest data possible and enable analysis of the full cancer pathway. These include, but are not limited to: Hospital Episode Statistics, Radiotherapy Dataset, Systematic Anti-Cancer Therapy Datatset and Routes to Diagnosis. This data will cover ten years preceding the start of the programme to provide a baseline and be routinely updated to assess changes throughout it.

All patients diagnosed with a first primary colorectal cancer (ICD10 C18-C20) in England from 1st January 2005 until the end of the study will be obtained from the National Cancer Registration and Analysis Service (NCRAS) [18] and assigned a managing MDT.

Direct PROMs and tissue collection

This research project is adopted by the NIHR Yorkshire and Humber Clinical Research Network and therefore recruitment will be undertaken across the region by network research and clinical staff working collaboratively. Recruitment will run over a 30 month period.

Eligible patients will be identified and approached in two ways.

1. Identified via the MDT and informed about the study by consultant letter sent out with their appointment letter for the primary pre-assessment clinic visit . Where possible, patients will be approached about the study at this clinic appointment. Patients missed at this appointment will be contacted at the earliest convenient time point and informed about the study.

2. Identified by their NHS clinical team if they present as an acute admission (for example with a bowel obstruction) and informed about the study by their clinical team following the emergency intervention (e.g. surgery).

Participants will have the option of completing the PROMs either online (ePROMs) using the University of Leeds secure questionnaire administration system QTool [19], accessed via the study website, (www.YCRBCIP.leeds.ac.uk) or on paper (pPROMs) with a pre-paid return envelope. They will be asked to complete the survey as soon as possible after consenting. This may be completed while attending hospital at the time of consent or later at home. Just prior to the 12 month follow-up questionnaire the patient status will be checked via NCRAS to confirm that the patient is still alive. Patients will then be sent an email/letter 12 months post-diagnosis by the YCR BCIP research team, inviting them to complete PROMs again (on paper or online according to patient preference). At both time points reminder letters or

For peer review only - http://bmjopen.bmj.com/site/about/guidelines.xhtml

emails will be sent at two weeks and followed up two weeks later (if no response) with the survey being resent with the reminder letter. The process is outlined in Figure 2.

The tissue samples to be collected for this study are:

1. excess pre-treatment, diagnostic biopsy tumour tissue
2. excess tissue following surgical resection (tumour and tumour-associated normal mucosal tissue)
3. excess tumour tissue following the biopsy or resection of distant metastases

The tissue samples will be formalin-fixed and paraffin wax-embedded as per the routine histopathology procedures at the patients' hospital. Tissue blocks will be used for any routine clinical diagnostic procedures required by the hospital before being sent to Pathology at the University of Leeds, and will be available to return to the hospital for further clinical testing if required.

Participants will be asked to complete a range of validated generic, cancer-specific and colorectal cancer-specific PROMs in addition to socio-demographic characteristics. Content has been informed by: clinical relevance, opinion of service users, overall length and participant burden[20], reviews of questionnaires measuring quality of life in colorectal cancer patients[21-25] and UK recommendations for the core outcomes set for trials in colorectal cancer surgery[26].

The survey comprises four sections at baseline and five sections at follow-up. The same PROMs are included at both time points. In addition, at the second time point (12-months post diagnosis) participants will be asked to complete questions about the financial costs of cancer. The time taken to complete the survey is estimated is about 30-35 minutes.

Section One: Your overall health and quality of life (both time points)

- European Organisation for Research and Treatment of Cancer (EORTC) Quality of Life Questionnaire QLQ-C30, the colorectal cancer specific module and items from the prostate, endometrial and cervical cancer modules [27-31]
- EuroQol Group EQ-5D-5L [32]

Section Two: Your everyday life and well-being (both time points)

- Social Difficulties Inventory (SDI-21) [33 34]
- The Short Warwick-Edinburgh Mental Well-being Scale (SWEMWBS) [35 36]

Section Three: Manging your health (both time points)

For peer review only - http://bmjopen.bmj.com/site/about/guidelines.xhtml

- Self-efficacy for Managing Chronic Disease [37]
- The Brief Illness Perceptions Questionnaire (B-IPQ) [38]

Section Four: The financial cost of cancer (Time 2 only)

- A questionnaire developed in-house based on one used in the ePOCS study[39].

Section Four: Questions about you (Time 1) and Section Five: Questions about you (Time 2)

Self-report socio-demographic and clinical details

Based on the number of diagnoses per year, there is potential to consent approximately 8,250 patients from sites across the region over a 30 month recruitment period. However due to eligibility restrictions, consent by MDT and by individuals, administrative/practical issues and staggered site recruitment start dates, the participation rate is likely to be less. Using estimates based on previous studies [40 41] the consent rate is estimated to be around 42% and the an attrition rate of approximately 35% at the 12 months follow-up point, giving a sample size of at least 1200. This will provide MDTs with a comprehensive descriptive profile of their patients in terms of quality of life outcomes; more complex analyses will be undertaken if sufficient numbers are accrued.

Audit and survey data

No additional patient-level data collection beyond that as part of routine patient care will be performed. For example, anonymised MRI scans may be used as part of an educational training initiative but these have already been performed as part of the patient care pathway. Clinicians at regional MDTs will complete surverys anonymously online using www.onlinesurveys.ac.uk (formally Bristol Online Survery). No specific recruitment will be performed for these data as only anonymised data used as part of routine patient care will be used. Regional MDT clinicians will be invited to complete the online surveys via email and through speciality group meetings.

Consent

Existing population-based datasets

The data is derived from patient-level information collected by the NHS, as part of the care and support of cancer patients. NCRAS has specific legal permission to collect this information without the need to seek consent, however, patients can ask NCRAS to remove their details from the cancer registry at any time. Access to cancer regristration data is controlled by the Public Health England Office for Data Release, and is only approved for

For peer review only - http://bmjopen.bmj.com/site/about/guidelines.xhtml

permitted medical purposes which include include: surveillance, clinical audit, service evaluation, ethically approved research, genetic counselling[42].

Direct PROMs and tissue collection

Identified patients will be provided with full study information (written and verbal) by specialist research nurses or Clinical Nurse Specialists who have undergone Good Clinical Practice (GCP training) to assure the rights, safety and wellbeing of research participants are protected[43]. Written consent may be taken at the time of this approach but patients will be given up to a week to think about study participation. Patients who wish to join the study will be asked to read, complete and sign a consent form, including their contact details name, address and/or email address. The person taking consent will also record the patient's date of birth, NHS number and gender. Patients must consent to both PROMs participation and tissue collection to be included in the study.

Patients are asked to consent to their clinical team being informed in the event that clinically relevant laboratory results are found. This includes the possibility of hereditary conditions identified through tumour testing e.g. Lynch syndrome or deficient mismatch repair status. These will be confirmed through routine NHS Clinical Genetics testing after counselling following referral from the local clinical teams with no germline testing taking place through the programme.

Audit and survey data

No specific consent will be needed for these data as only anonymised data used as part of routine patient care will be used, for example specimen photographs or scans will have all identifiers removed.

Data Linkage

Whilst intitally the existing population datasets and direct PROMs and tissue collection will be analysed separately, these will subsequently be linked together together through the UK Bowel Cancer Intelligence Hub using name, NHS number and date of birth. This will provide additional patient characterisitcs for analysis of the PROMs and tissue data.

Analysis

Existing population-based datasets

Baseline assessments of care in the region are to be performed on individuals diagnosed with colorectal cancer (ICD10 C18-C20) in Yorkshire and Humber, enabling comparison

For peer review only - http://bmjopen.bmj.com/site/about/guidelines.xhtml

between teams in the region and with national data[16]. Initially this includes using descriptive analysis and statistical methods such as regression modelling, survival analysis and funnel plots [44] comparing the following data: demographic characteristics, tumour characteristics, surgery and oncology management and short and long-term outcomes.

Some analyses will be rerun periodically over the course of the programme to evaluate the impact on outcomes of specific educational interventions. The measures to be analysed and the sources of these can be found in Table 1.

Direct PROMs and tissue collection

Descriptive statistics will be used to report the survey results and assess the quality of life outcomes of the participants. Following data linkage, the outcomes will be analysed according to stage of disease, treatment type, comorbidity, age, ethnic and sociodemographic group (and other relevant variables). These descriptive analyses will identify potential relationships of interest which can be investigated further. Regression modelling will be used to investigate associations amongst the different types of variables to identify statistically and clinically significant risk factors and predictors of outcomes. In order to be robust, analyses will require appropriate adjustment for case-mix and other confounding factors and may require more complex techniques, such as the modelling of hierarchies within the data (multilevel modelling), post-hoc weighting to overcome response bias and multiple imputation of missing data.

Tissue samples removed at surgery or biopsy which are surplus to routine clinical requirements will be utilised by the research team for upfront testing of novel biomarkers. YCR BCIP will test for novel or rare potentially treatable targets via a range of molecular techniques. The immunohistochemical markers of interest include, HER2, PTEN, PD-1 and PD-L1, amphiregulin and epiregulin, and immune system markers CD3 and CD8. The study will also undertake phenotype analysis by using high-resolution scanned images of tumours using novel algorithms to identify improved prognostic and predictive markers of outcome.

Next Generation Sequencing and/or pyrosequencing will be performed on any extracted DNA to identify tumours with specific gene hotspot mutations including, KRAS, NRAS, BRAF, EGFR and PIK3CA. Other molecular markers linked to causation, outcome and response to therapy will also be investigated e.g. bacterial toxin carriage or fusobacterium nucleatum.

For peer review only - http://bmjopen.bmj.com/site/about/guidelines.xhtml

Analysis of RNA expression levels of receptor ligands such as amphiregulin and epiregulin, and HER2 and HER3 may also be performed.

Ultimately, the identification of treatable molecular markers will allow treatment to be targeted to specific tumour types in an effort to improve outcomes, benefitting future patients. Some participating patients may directly benefit from the stufy by being referred to a clinical trial, giving them the chance to receive tumour-specific treatment which is not routinely available.

Audit and survey data

The analyses of audit and survery data will be dependent clinical specialities involved and the nature of the data collected. For example, the completion of MRI scans for rectal cancer at an educational training initiative would be assessed by agreement cofficients and results of surveys will be analysed using descriptive statistics.

Data Safeguards

Participant recruitment will be undertaken by each Trust involved in the study. Each research network site will allocate a study ID for potential participants. They will use a University of Leeds secure electronic transfer system every two weeks to inform the YCR BCIP research team of all recruitment activity. This will include the consented patients contact details, date of birth and NHS number to allow for follow-up and to ensure tissue blocks are appropriately labelled for tracking and data linkage. All subsequent participant contact will be undertaken by the central YCR BCIP team.

Storage of all hard copy documents will be in locked metal filing cabinets in research offices of the University of Leeds with secure access building controls. Tissue samples will be used and stored in a scure building with restricted access. Electronic data with pseudonomysed (allocated ID number) patient information will be stored in a secure environment. These files will only be accessible to relevant members of the study analysis team. Where temporary storage of sensitive data is required (e.g. contact details for sending out repeat surveys), files will be accessible only to relevant members of the research team and not stored with any linked data. Members of the analysis team will not have access to any identifiable data. Hard copy data will be kept for 5 years following the end of the study (until 2026) for long term follow-up.

Development of Educational Interventions

For peer review only - http://bmjopen.bmj.com/site/about/guidelines.xhtml

The analyses will be disseminated and discussed by regional MDTs at events for each clinical discipline. Agreements of educational needs to improve care will be agreed along with any additional data capture and audit processes that teams agree are required. The process is repeated with ongoing data analyses to establish the improvements in management and outcomes that have occurred (Figure 1). As YCR BCIP progresses, further work will be undertaken looking at other outcomes including, but not limited to, those related to screening, pathology and long-term outcomes.

Public Patient Involvement

Patients and carers were actively engaged through the PROMS Working Group to develop the design and content of the patient questionnaires, the patient information sheet and consent form. At the request of the patients and carers, additional questions were included around the financial impact of cancer and a specific request was made to EORTC to amend the EORTC colorectal module and add specific questions from other EORTC modules to understand side effects of cancer and cancer treatments. EORTC granted these specific amendments for this study. The electronic and paper copies of the final draft questionnaires were tested with patients attending a colorectal cancer follow-up clinic at one of the region hospitals. Modification to the layout of the questionnaires were made following the results of the testing. The testing gave an understanding of the length of time it took patients to complete the questionnaires. The PROMS Working Group will remain active throughout the length of the study; the group will be kept apprised of recruitment levels and early results. It is expected that the patients will advise on the analysis and how the results are communicated to the regional clinical teams and wider audiences.

For peer review only

Discussion

The YCR BCIP study ambitiously aims to improve outcomes for patients with colorectal cancer across the Yorkshire and Humber region by in-depth analysis of existing and newly captured data, and by actively engaging local MDTs. In its initial stages, this will be done by demonstrating the variation in the demographics, management and outcomes in the region using routine NHS datasets. However, given the limitations of what can be achieved with existing data, the YCR BCIP is collecting additional data to analyse alongside this with the purpose to better understand what is driving the observed variation.

For peer review only - http://bmjopen.bmj.com/site/about/guidelines.xhtml

The PROMs data will enrich other study data and allow for an ‘in-depth’ description of what life is really like for colorectal cancer patients at diagnosis and a year later. At present, the patient voice is not ‘measured’ routinely as part of clinical practice, neglecting the impact of illness and treatment on the everyday lives of patients. YCR BCIP will change this with the integration of PROMs administration into clinical practice[41]. The PROMs used in this study have been selected with input from experts in colorectal cancer and psychosocial care: patients, clinical nurse specialists and doctors. Although the length of the survey would not be feasible to administer in everyday practice, information on which PROMs provide the most meaningful data will be obtained. In the future risk-stratified follow-up may incorporate not only clinical indicators but also key quality of life indicators to inform best supportive care[45].

Results from the tissue collection and testing could impact on treatment and follow-up decisions for the participating patients and, potentially, their families. For example, the results may indicate that a patient could benefit from a targeted treatment being tested through an open clinical trial if they develop an indication for further treatment e.g. Medical Research Council FOCUS4 [46]. Increased risks of having a hereditary condition may be identified, which not only has implications for the patient but also their family.

The YCR BCIP is offering a novel approach to address the variation in colorectal cancer care across a large region of the UK. Engagement of the region’s MDTs with their data will lead to a range of educational initiatives, studies and clinical audits that aim to optimise practice across the region. It is planned that the outcomes of these will be presented to the relevant specialty group for review and to develop actions based on findings. The main limiting factor for the success of the study is that to understand the overall picture of colorectal cancer care and the ability to improve this in the region, relies on the extent of engagement from MDTs.

Ethics and dissemination

The study was granted ethical approval (17/WM/0374) by the West Midlands – Solihull Research Ethics Committee in December 2017. The study was approved by the Health Research Authority and granted approval for inclusion in the National Institute for Health Research’s portfolio of studies in December 2017 (Project ID 227673). Further work from

For peer review only - http://bmjopen.bmj.com/site/about/guidelines.xhtml

the seven specialty groups involved in the programme: surgery, radiology, pathology, clinical oncology, medical oncology, nursing and anaesthetics, will be reported as publications soon.

Authors' contributions

PQ is the principal investigator.NW, PW and EM are coinvestigators and/or workstream leads. Together these authors conceived and designed the study. HR and JM manage the study. JT provides statistical support. AG provides pathology support. All authors contributed to writing of the manuscript and have approved a final version.

Competing interests

The authors declare that they have no competing interests.

Funding

This work was funded by Yorkshire Cancer Research (Award reference number L394). Prof Quirke holds an NIHR Senior Investigator award. The UK Bowel Cancer Intelligence Hub is supported by Cancer Research UK (C23434/A23706).

Acknowledgements

The YCR BCIP study group includes Philip Quirke, Paul Finan, Penny Wright, Nicholas West, Matthew Seymour, Eva Morris, David Sebag-Montefoire, Daniel Swinson, Damian Tolan, Simon Howell, Peter Brown, John Taylor, Amy Glover, Aidan Hindley, Hannah Rossington, Jackie Mara. The YCR BCIP research team acknowledges the support of the National Institute for Health Research Clinical Research Network (NIHR CRN).

References

1. Allemani C, Matsuda T, Di Carlo V, et al. Global surveillance of trends in cancer survival 2000–14 (CONCORD-3): analysis of individual records for 37 513 025 patients diagnosed with one of 18 cancers from 322 population-based registries in 71 countries. *The Lancet* 2018 doi: 10.1016/s0140-6736(17)33326-3
2. Heald RJ, Ryall RD. Recurrence and survival after total mesorectal excision for rectal cancer. *Lancet* 1986;1(8496):1479-82. [published Online First: 1986/06/28]
3. Iversen LH, Green A, Ingeholm P, et al. Improved survival of colorectal cancer in Denmark during 2001-2012 - The efforts of several national initiatives. *Acta Oncol* 2016;55 Suppl 2:10-23. doi: 10.3109/0284186X.2015.1131331
4. Holm T, Johansson H, Cedermark B, et al. Influence of hospital- and surgeon-related factors on outcome after treatment of rectal cancer with or without preoperative radiotherapy. *Br J Surg* 1997;84(5):657-63.

For peer review only - http://bmjopen.bmj.com/site/about/guidelines.xhtml

5. Wibe AM, B.; Norstein, J.; Carlsen, E.; Wiig, J.N.; Heald, R.J.; Langmark, F.; Myrvold, H.E.; Soreide, O.; for the Norwegian Rectal Cancer Group. A National Strategic Change in Treatment Policy for Rectal Cancer - Implementation of Total Mesorectal Excision as Routine Treatment in Norway. A National Audit. *Dis Colon Rectum* 2002;45(7):857-66. doi: 10.1097/01.Dcr.0000019492.53207.70
6. Iversen LH, Ingeholm P, Gogenur I, et al. Major reduction in 30-day mortality after elective colorectal cancer surgery: a nationwide population-based study in Denmark 2001-2011. *Ann Surg Oncol* 2014;21(7):2267-73. doi: 10.1245/s10434-014-3596-7
7. Martling A, Holm T, Rutqvist LE, et al. Impact of a surgical training programme on rectal cancer outcomes in Stockholm. *Br J Surg* 2005;92(2):225-9. doi: 10.1002/bjs.4834
8. Martling AL, Holm T, Rutqvist LE, et al. Effect of a surgical training programme on outcome of rectal cancer in the County of Stockholm. *The Lancet* 2000;356(9224):93-96. doi: 10.1016/s0140-6736(00)02469-7
9. Moran BJ, Holm T, Brannagan G, et al. The English national low rectal cancer development programme: key messages and future perspectives. *Colorectal Dis* 2014;16(3):173-8. doi: 10.1111/codi.12501
10. Morris EJ, Finan PJ, Spencer K, et al. Wide Variation in the Use of Radiotherapy in the Management of Surgically Treated Rectal Cancer Across the English National Health Service. *Clin Oncol (R Coll Radiol)* 2016;28(8):522-31. doi: 10.1016/j.clon.2016.02.002
11. Vallance AE, van der Meulen J, Kuryba A, et al. The timing of liver resection in patients with colorectal cancer and synchronous liver metastases: a population-based study of current practice and survival. *Colorectal Dis* 2018;20(6):486-95. doi: 10.1111/codi.14019
12. Allemani C, Matsuda T, Di Carlo V, et al. Global surveillance of trends in cancer survival 2000-14 (CONCORD-3): analysis of individual records for 37 513 025 patients diagnosed with one of 18 cancers from 322 population-based registries in 71 countries. *Lancet* 2018 doi: 10.1016/S0140-6736(17)33326-3
13. YCR. Yorkshire Cancer Research; [Available from: https://yorkshirecancerresearch.org.uk/.
14. Jessop J, Beagley C, Heald RJ. The Pelican Cancer Foundation and The English National MDT-TME Development Programme. *Colorectal Dis* 2006;8 Suppl 3:1-2. doi: 10.1111/j.1463-1318.2006.01060.x
15. Wibe A, Moller B, Norstein J, et al. A national strategic change in treatment policy for rectal cancer--implementation of total mesorectal excision as routine treatment in Norway. A national audit. *Dis Colon Rectum* 2002;45(7):857-66.
16. Abstracts of the Association of Coloproctology of Great Britain and Ireland Annual Meeting, 3-5 July 2017, Bournemouth, UK. *Colorectal Dis* 2017;19 Suppl 4:3-66. doi: 10.1111/codi.13849
17. UK Bowel Cancer Intelligence Hub https://bci.leeds.ac.uk/: University of Leeds; 2018 [Available from: https://bci.leeds.ac.uk/ accessed 050218 2018.
18. Public Health England. National Cancer Registration and Analysis Service http://www.ncr.nhs.uk/: Public Health England; 2015 [Available from: http://www.ncr.nhs.uk/ accessed 27/10/2015 2015.
19. Ashley L, Jones H, Thomas J, et al. Integrating cancer survivors' experiences into UK cancer registries: design and development of the ePOCS system (electronic Patient-reported Outcomes from Cancer Survivors). *Br J Cancer* 2011;105 Suppl 1:S74-81. doi: 10.1038/bjc.2011.424

For peer review only - http://bmjopen.bmj.com/site/about/guidelines.xhtml

20. Foster C, Haviland J, Winter J, et al. Pre-Surgery Depression and Confidence to Manage Problems Predict Recovery Trajectories of Health and Wellbeing in the First Two Years following Colorectal Cancer: Results from the CREW Cohort Study. *Plos One* 2016;11(5) doi: 10.1371/journal.pone.0155434
21. Ganesh V, Agarwal A, Popovic M, et al. Comparison of the FACT-C, EORTC QLQ-CR38, and QLQ-CR29 quality of life questionnaires for patients with colorectal cancer: a literature review. *Supportive Care in Cancer* 2016;24(8):3661-68. doi: 10.1007/s00520-016-3270-7
22. Gilbert A, Sebag-Montefiore D, Davidson S, et al. Use of patient-reported outcomes to measure symptoms and health related quality of life in the clinic. *Gynecologic Oncology* 2015;136(3):429-39. doi: 10.1016/j.ygyno.2014.11.071
23. Hadi M, Gibbons E, Fitzpatrick R. A structured review of patient reported outcome measures for colorectal cancer. In: Patient Reported Outcome Measurement Group UoO, ed. Patient Reported Outcome Measures. Oxford, 2010.
24. McNair AGK, Whistance RN, Forsythe RO, et al. Synthesis and summary of patient-reported outcome measures to inform the development of a core outcome set in colorectal cancer surgery. *Colorectal Dis* 2015;17(11):O217-O29. doi: 10.1111/codi.13021
25. Wong CKH, Chen J, Yu CLY, et al. Systematic review recommends the European Organization for Research and Treatment of Cancer colorectal cancer-specific module for measuring quality of life in colorectal cancer patients. *Journal of Clinical Epidemiology* 2015;68(3):266-78. doi: 10.1016/j.jclinepi.2014.09.021
26. McNair AG, Whistance RN, Forsythe RO, et al. Core Outcomes for Colorectal Cancer Surgery: A Consensus Study. *PLoS Med* 2016;13(8):e1002071. doi: 10.1371/journal.pmed.1002071
27. Aaronson NK, Ahmedzai S, Bergman B, et al. The European Organization for Research and Treatment of Cancer QLQ-C30: a quality-of-life instrument for use in international clinical trials in oncology. *Journal of the National Cancer Institute* 1993;85(5):365-76.
28. Greimel E, Nordin A, Lanceley A, et al. Psychometric validation of the European Organisation for Research and Treatment of Cancer Quality of Life Questionnaire-Endometrial Cancer Module (EORTC QLQ-EN24). *Eur J Cancer* 2011;47(2):183-90. doi: 10.1016/j.ejca.2010.08.014
29. Greimel ER, Kuljanic Vlasic K, Waldenstrom AC, et al. The European Organization for Research and Treatment of Cancer (EORTC) Quality-of-Life questionnaire cervical cancer module: EORTC QLQ-CX24. *Cancer* 2006;107(8):1812-22. doi: 10.1002/cncr.22217
30. van Andel G, Bottomley A, Fossa SD, et al. An international field study of the EORTC QLQ-PR25: a questionnaire for assessing the health-related quality of life of patients with prostate cancer. *Eur J Cancer* 2008;44(16):2418-24. doi: 10.1016/j.ejca.2008.07.030
31. Whistance RN, Conroy T, Chie W, et al. Clinical and psychometric validation of the EORTC QLQ-CR29 questionnaire module to assess health-related quality of life in patients with colorectal cancer. *Eur J Cancer* 2009;45(17):3017-26. doi: 10.1016/j.ejca.2009.08.014
32. Herdman M, Gudex C, Lloyd A, et al. Development and preliminary testing of the new five-level version of EQ-5D (EQ-5D-5L). *Qual Life Res* 2011;20(10):1727-36. doi: 10.1007/s11136-011-9903-x

For peer review only - http://bmjopen.bmj.com/site/about/guidelines.xhtml

33. Wright EP, Kiely M, Johnston C, et al. Development and evaluation of an instrument to assess social difficulties in routine oncology practice. *Qual Life Res* 2005;14(2):373-86.
34. Wright P, Smith AB, Keding A, et al. The Social Difficulties Inventory (SDI): development of subscales and scoring guidance for staff. *Psychooncology* 2011;20(1):36-43. doi: 10.1002/pon.1705
35. Tennant R, Hiller L, Fishwick R, et al. The Warwick-Edinburgh Mental Well-being Scale (WEMWBS): development and UK validation. *Health Qual Life Outcomes* 2007;5:63. doi: 10.1186/1477-7525-5-63
36. Bartram DJ, Sinclair JM, Baldwin DS. Further validation of the Warwick-Edinburgh Mental Well-being Scale (WEMWBS) in the UK veterinary profession: Rasch analysis. *Qual Life Res* 2013;22(2):379-91. doi: 10.1007/s11136-012-0144-4
37. Lorig KR, Sobel DS, Ritter PL, et al. Effect of a self-management program on patients with chronic disease. *Eff Clin Pract* 2001;4(6):256-62.
38. Broadbent E, Petrie KJ, Main J, et al. The brief illness perception questionnaire. *J Psychosom Res* 2006;60(6):631-7. doi: 10.1016/j.jpsychores.2005.10.020
39. Marti J, Hall PS, Hamilton P, et al. The economic burden of cancer in the UK: a study of survivors treated with curative intent. *Psychooncology* 2016;25(1):77-83. doi: 10.1002/pon.3877
40. Ashley L, Jones H, Thomas J, et al. Integrating patient reported outcomes with clinical cancer registry data: a feasibility study of the electronic Patient-Reported Outcomes From Cancer Survivors (ePOCS) system. *J Med Internet Res* 2013;15(10):e230. doi: 10.2196/jmir.2764
41. Black N, Burke L, Forrest CB, et al. Patient-reported outcomes: pathways to better health, better services, and better societies. *Qual Life Res* 2016;25(5):1103-12. doi: 10.1007/s11136-015-1168-3
42. ODR. Public Health England Office for Data Release; [Available from: https://www.gov.uk/guidance/national-cancer-registration-and-analysis-service-ncras - access-to-cancer-registration-data.
43. NIHR. National Institute for Health Research; [Available from: https://www.nihr.ac.uk/health-and-care-professionals/learning-and-support/good-clinical-practice.htm.
44. Spiegelhalter DJ. Funnel plots for comparing institutional performance. *Stat Med* 2005;24(8):1185-202. doi: 10.1002/sim.1970
45. Watson EK, Rose PW, Neal RD, et al. Personalised cancer follow-up: risk stratification, needs assessment or both? *Br J Cancer* 2012;106(1):1-5. doi: 10.1038/bjc.2011.535
46. Kaplan R, Maughan T, Crook A, et al. Evaluating many treatments and biomarkers in oncology: a new design. *J Clin Oncol* 2013;31(36):4562-8. doi: 10.1200/JCO.2013.50.7905

Figure Legends

Figure 1: Yorkshire Cancer Research Bowel Cancer Improvement Programme (YCRBCIP) process and study design.

For peer review only - http://bmjopen.bmj.com/site/about/guidelines.xhtml

Figure 2: Process for the Patient Reported Outcomes Measures (PROMs) study.

For peer review only

For peer review only - http://bmjopen.bmj.com/site/about/guidelines.xhtml

	Data Source		
	**Existing population-based datasets**	**Direct PROMs and tissue collection**	**Audit and survey data**
**Patient and Tumour Characteristics**			
Age & sex	Yes (NCRAS)	Yes	
Ethnicity	Yes (NCRAS)	Yes	
Height & Weight		Yes	
Comorbidity	Yes (NCRAS)	Yes	
Socio-economic status	Yes (NCRAS)	Yes	
Stage & site	Yes (NCRAS)		
Method of admission	Yes (RtD)		
**Treatment Variation**			
Surgical resection rate	Yes (HES)		
Quality of surgery			Yes
Abdominoperineal excision rate	Yes (HES)		
Use of adjuvant & palliative chemotherapy	Yes (SACT)		Yes
Use of neoadjuvant radiotherapy	Yes (RTDS)		
Use of laparoscopic surgery	Yes (HES)		
Emergency care procedures	Yes (HES)		Yes
Practice of anaesthetics			Yes
Quality of MRI reporting			Yes
Quality of CT imaging			Yes
Liver metastases resection rate	Yes (HES)		
Nodal yields and retrieval methods	Yes (NCRAS)		Yes
**Outcomes**			
30-day Postoperative mortality	Yes (NCRAS)		
1 to 5 year overall and net survival	Yes (NCRAS)		
18-month postoperative stoma rate	Yes (HES)		
Postoperative hospital stay	Yes (HES)		
Emergency readmission rates	Yes (HES)		
Overall health and quality of life		Yes	
Everyday life and well-being		Yes	
Self-efficacy for Managing Chronic Disease		Yes	
Financial cost of cancer		Yes	
Urinary function and faecal incontinence		Yes	
Sexual functioning		Yes	
Lower anterior resection syndrome		Yes	
Molecular subtyping		Yes	

Abbreviations: Magnetic resonance imaging (MRI), Computerized Tomography (CT), National Cancer Registration and Analysis Service (NCRAS), Routes to Diagnosis (RtD), Hospital Episode Statistics (HES), Systemic Anti-Cancer Therapy Dataset (SACT), Radiotherapy Dataset (RTDS).

Table 1. Measures of patient characteristics, treatment measures and outcomes to analysed.

For peer review only - http://bmjopen.bmj.com/site/about/guidelines.xhtml

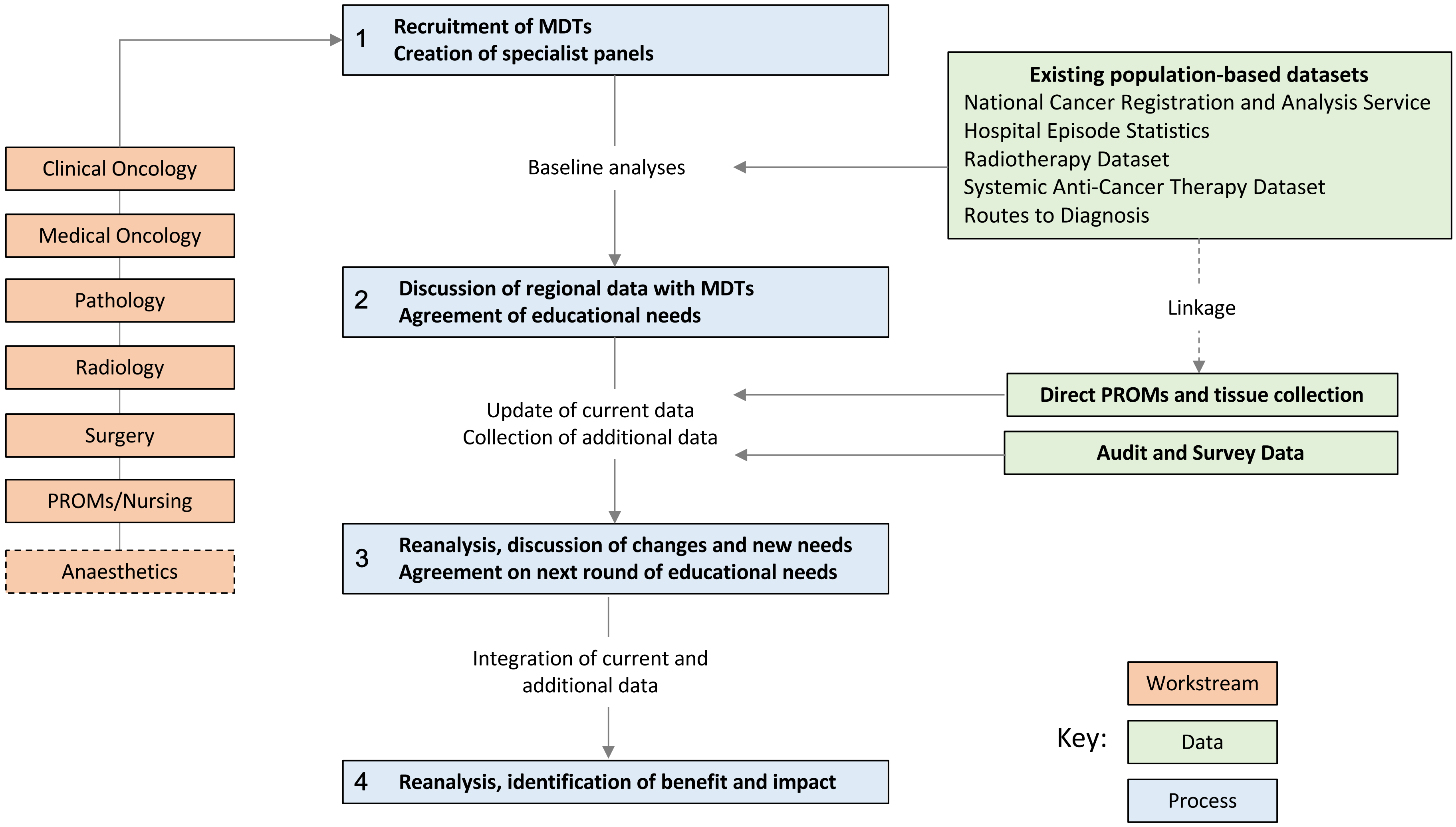
1 Recruitment of MDTs
Creation of specialist panels
Existing population-based datasets
National Cancer Registration and Analysis Service
Hospital Episode Statistics
Radiotherapy Dataset
Systemic Anti-Cancer Therapy Dataset
Routes to Diagnosis
Clinical Oncology
Medical Oncology
Pathology
Radiology
Surgery
PROMs/Nursing
Anaesthetics
Baseline analyses
2 Discussion of regional data with MDTs
Agreement of educational needs
Linkage
Direct PROMs and tissue collection
Audit and Survey Data
Update of current data
Collection of additional data
3 Reanalysis, discussion of changes and new needs
Agreement on next round of educational needs
Integration of current and
additional data
4 Reanalysis, identification of benefit and impact
Key:
Workstream
Data
Process

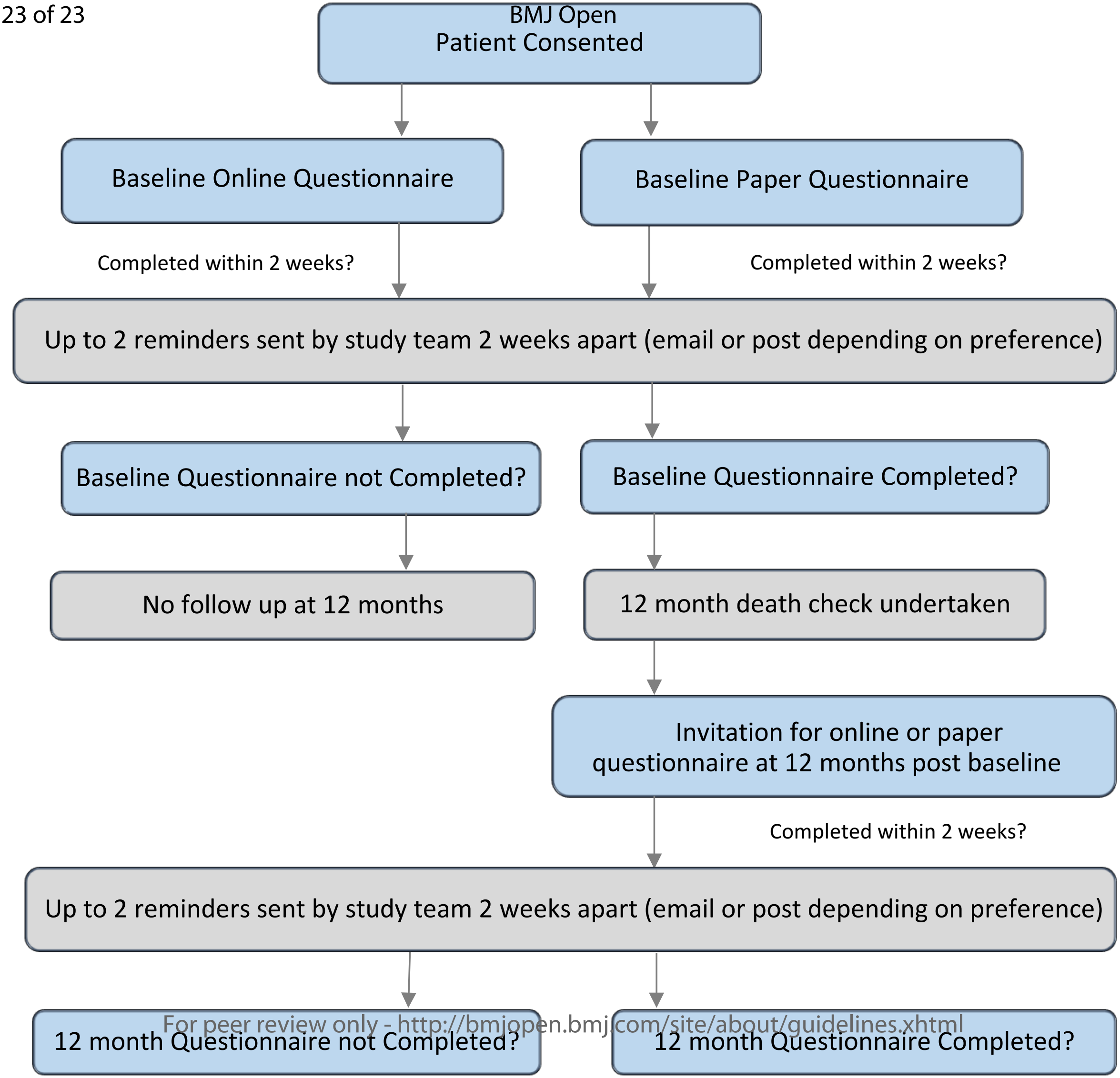

For peer review only - http://bmjopen.bmj.com/site/about/guidelines.xhtml

BMJ Open

A regional multidisciplinary team intervention programme to improve colorectal cancer outcomes: study protocol for the Yorkshire Cancer Research Bowel Cancer Improvement Programme (YCR BCIP)

Journal:	*BMJ Open*
Manuscript ID	bmjopen-2019-030618.R3
Article Type:	Protocol
Date Submitted by the Author:	21-Oct-2019
Complete List of Authors:	Taylor, John; Leeds Institute of Cancer and Pathology, Section of Epidemiology and Biostatistics, University of Leeds<br>Wright, Penny; Leeds Institute of Cancer and Pathology, Section of Patient Centred Outcome Research, University of Leeds<br>Rossington, Hannah; Leeds Institute of Cancer and Pathology, Section of Epidemiology and Biostatistics, University of Leeds<br>Mara, Jackie; Leeds Institute of Cancer and Pathology, Section of Epidemiology and Biostatistics, University of Leeds<br>Glover, Amy; Leeds Institute of Cancer and Pathology, Section of Pathology and Tumour Biology, University of Leeds<br>West, Nick; Leeds Institute of Cancer and Pathology, Section of Pathology and Tumour Biology, University of Leeds<br>Morris, Eva; Leeds Institute of Cancer and Pathology, Section of Epidemiology and Biostatistics, University of Leeds<br>Quirke, Phillip; Leeds Institute of Cancer and Pathology, Section of Pathology and Tumour Biology, University of Leeds
**Primary Subject Heading**:	Epidemiology
Secondary Subject Heading:	Health policy, Medical education and training, Oncology, Research methods
Keywords:	Quality in health care < HEALTH SERVICES ADMINISTRATION & MANAGEMENT, Gastrointestinal tumours < ONCOLOGY, PUBLIC HEALTH, STATISTICS & RESEARCH METHODS, EPIDEMIOLOGY

SCHOLARONE™
Manuscripts

A regional multidisciplinary team intervention programme to improve colorectal cancer outcomes: study protocol for the Yorkshire Cancer Research Bowel Cancer Improvement Programme (YCR BCIP)

J.C. Taylor[1*], P. Wright[2], H.L. Rossington[1], J. Mara[1], A. Glover[3], N.P. West[3], E.J.A. Morris[1], P. Quirke[3] on behalf of YCR BCIP Study Group.

1. Cancer Epidemiology Group, Section of Epidemiology and Biostatistics, Leeds Institute of Cancer & Pathology and Leeds Institute for Data Analytics, University of Leeds, Leeds, UK
2. Section of Patient Centred Outcome Research, Leeds Institute of Cancer & Pathology, University of Leeds, Leeds, UK
3. Section of Pathology, Leeds Institute of Medical Research at St. James's, University of Leeds, Leeds, UK

Corresponding author:

J. C. Taylor

Leeds Institute for Data Analytics

Worsley Building

University of Leeds

LS2 9NL, Leeds, UK

j.c.taylor@leeds.ac.uk

For peer review only

For peer review only - http://bmjopen.bmj.com/site/about/guidelines.xhtml

Abstract

Introduction

Although colorectal cancer outcomes in England are improving they remain poorer than many comparable countries. Yorkshire Cancer Research have, therefore, established a Bowel Cancer Improvement Programme (YCR BCIP) to improve colorectal cancer outcomes within Yorkshire and Humber, a region representative of the nation. It aims to do this by quantifying variation in practice, engaging with the colorectal multidisciplinary teams (MDTs) to understand this and developing educational interventions to minimise it and improve outcomes.

Methods and analysis

Initially, routine health datasets will be used to quantify variation in the demographics, management and outcomes of patients across the Yorkshire and Humber region and results presented to MDTs. The YCR BCIP is seeking to supplement these existing data with patient reported health-related quality of life information (PROMs) and tissue sample analysis. Specialty groups (surgery, radiology, pathology, clinical oncology, medical oncology, clinical nurse specialists and anaesthetics) have been established to provide oversight and direction for their clinical area within the programme, to review data and analysis and to develop appropriate educational initiatives.

Ethics and dissemination

The YCR BCIP is aiming to address the variation in practice to significantly improve colorectal cancer outcomes across the Yorkshire and Humber region. PROMs and tissue sample collection and analysis will help to capture the information required to fully assess care in the region. Engagement of the region's MDTs with their data will lead to a range of educational initiatives, studies and clinical audits that aim to optimise practice across the region.

For peer review only - http://bmjopen.bmj.com/site/about/guidelines.xhtml

Stengths and limitations of this study

- This study uses a novel approach by providing regional colorectal cancer MDTs with data and, with their input, developing educational interventions to minimise any variation seen in order to optimise outcomes.
- The strength of this study includes evaluating variation in the management and outcomes of colorectal cancer patients by combining routinely collected clinical datasets and novel information on aspects of care not currently quantifiable through existing datasets.
- The study will facilitate implementation of guidelines such as routine screening for Lynch syndrome and deficient mismatch repair status.
- The potential of the study is limited by the extent of engagement from regional colorectal cancer MDTs.

For peer review only

For peer review only - http://bmjopen.bmj.com/site/about/guidelines.xhtml

Introduction

Colorectal cancer is a common disease in the UK with over 41,000 people diagnosed each year and 16,000 people dying from it. Although survival rates have improved over time, they continue to lag behind those attained by many economic neighbours[1] and so developing educational interventions to improve outcomes is a priority.

Some potential explanations for these survival differences include quality of care and access to services. For example, local recurrence and survival rates in rectal cancer are related to the quality of the surgical resection[2] so improving surgical quality is likely to improve outcomes. Likewise, ensuring gold standard diagnostic, staging and multidisciplinary care across all aspects of the patient pathway have a demonstrable impact on outcomes[3] while screening for Lynch syndrome and deficient mismatch repair will enable more effective managemement of patients.

Other countries have acknowledged these relationships and taken steps to improve standards. For instance, in the early 1990s, Norway recognised there was huge variation in local recurrence rates depending on which surgeon operated[4]. In response, they initiated a training programme to quantify variation and improve the quality of surgery. This strategy was a success, reducing local recurrence and improving overall survival[5]. As a result similar programmes in other countries, including the UK were established with equally positive outcomes including lower postoperative mortality[6] and better survival[3 7], lower permanent stoma[7] and local recurrence rates[8], better preoperative staging[9], improved selection of patients for non-surgical treatment[9] and the need for less emergency surgery[3].

Whilst these programmes have undoubtedly had a positive impact, variations in management remain and particularly so in the UK[10 11]. Although survival rates in the UK have improved, the gap to rates attained in comparable countries[12] have not closed. A better understanding of what is driving these variations will help to target educational interventions to improve care.

Yorkshire Cancer Research is a charity whose aim is to ensure that more people of Yorkshire and Humber "avoid, survive and cope" with cancer and, by 2025, they intend to have reduced cancer deaths in the region by 2,000 per year[13]. As part of their strategy to achieve this they have recently funded the Bowel Cancer Improvement Programme (YCR BCIP). This

For peer review only - http://bmjopen.bmj.com/site/about/guidelines.xhtml

initiative builds upon the experience gained from English National programmes [9 14] and extends the approach of those successful programmes completed in Scandinavia [3 8 15]. It centres on the collection and analysis of robust colorectal cancer data to examine practice across the region, ensuring the multidisciplinary teams (MDTs) managing the disease engage with these data and agree on areas for improvement. Educational interventions or other strategies can then be developed and implemented to ensure optimal practice is achieved.

This protocol paper gives an overview the YCR BCIP study, which has the following aims:

1. Quantify and report the variation in the demographics, management and outcomes of the region's colorectal cancer patients using
    a. routinely collected clinical datasets,
    b. new information on aspects of care not currently quantifiable through existing datasets (e.g. Patient Reported Outcome Measures (PROMs), the molecular subtyping of the disease and the quality of radiology, pathology and surgery).
2. Determine how outcomes from the Yorkshire and Humber region compare to the rest of England.
3. Provide the Yorkshire and Humber region colorectal MDTs with these data and, with their input, develop educational interventions to minimise any variation seen in order to optimise outcomes.
4. Facilitate implementation of guidelines such as routine screening for Lynch syndrome and deficient mismatch repair status.
5. Evaluate improvement in outcomes over the study period.

For peer review only - http://bmjopen.bmj.com/site/about/guidelines.xhtml

Methods and analysis

Setting

The Yorkshire and Humber region of the UK has a population of approximately 5.7 million with around 3,300 colorectal cancer diagnoses a year, amounting to just over 10% of diagnoses in England. Patients diagnosed with colorectal cancer in the region are managed by 16 different MDTs. These operate across 14 National Health Service (NHS) Trusts which provide health services for particular geographical areas of the region which exhibit a range of workloads, ranging between approximately 120-390 cases a year (4%-12% of the regional workload). Patients within the geographical areas covered by each Trust exhibit a variable range of demographic features such as socioeconomic deprivation and comorbidity [16].

Programme Overview

YCR BCIP will examine routinely collected NHS data and, subject to patient consent, additional data collected specifically for the programme. Baseline performance outcomes for each MDT from across the region will be assessed. The knowledge will be fed back to each MDT and a programme of educational interventions developed based on the performance outcomes. The process will be iterative as outlined in Figure 1. The programme commenced on 1st April 2016 and will run until 31 March 2021.

Following a regional meeting of MDT representatives to discuss, plan and agree to programme participation held in June 2016, six specialty groups were initially established. The remit of these groups is to provide oversight and direction for their clinical specialty within the programme, to review data and analyses and to develop appropriate educational initiatives. The six groups focused on the main clinical disciplines involved in the MDT; surgery, radiology, pathology, clinical oncology, medical oncology, and specialist nursing. Group membership was drawn from all of the region's MDTs and an individual identified from each to to act as a coordinator between the research team and MDT. The need for a seperate anaesthetics workstream was subsequently identified and introduced. A lead clinician was also identified for each discipline, having the responsibility to coordinate educational events, gather opinion on best practice, formulate consensus views and drive agreed initiatives into routine clinical care across the region.

For peer review only - http://bmjopen.bmj.com/site/about/guidelines.xhtml

Data Sources

Although broad ranging, it was recognised that the routine colorectal cancer data available is not sufficient to quantify all aspects of care robustly. Whilst routine data provides good information available on 'hard' outcomes such as which operation was undertaken or survival time, there is a lack of information on what this really means to the individual concerned and the quality of that survival. This precludes the production of the rich intelligence the MDTs want to fully understand variation in care. Therefore data collated in YCR BCIP will come from three main sources:

Existing population-based datasets

Consisting of routine NHS datasets providing both regional data (around 3,300 cases per year) and comparative national data (around 28,700 cases per year) on patient and tumour characteristics, treatment choices, diagnosis pathways and patient outcomes. All patients diagnosed with a first primary colorectal cancer (ICD10 C18-C20) in England from 1st January 2005 until the end of the study and registered in the National Cancer Registration and Analysis Service (NCRAS)[17] will be eligible.

Direct PROMs and tissue collection

Consisting of regional PROMs data collected directly from patients via a questionnaire on health-related quality of life (at both the time of diagnosis, before primary treatment if possible and again at 12 months post diagnosis) and molecular testing of tumour and tumour-associated normal mucosal tissue samples.

All MDTs in the region have been invited to participate in this element of the YCR BCIP. Every colorectal cancer patient (ICD10 C18-C20) is eligible if they are considered suitable for treatment, English language literate, aged at least 18 years old and with capacity to give informed consent. Participants will be asked to consent to the study prior to commencement of primary treatment or following an emergency intervention.

Audit and survey data

Consisting of unlinked anonymised data collected from clinicians at regional MDTs in the form of an audit or a clinician survey. The exact nature of these shall be identified by each clinical discipline depending on the needs and availability of existing data. However, they are expected to include but are not limited to: an audit on surgical quality using specimen photographs, audits assessing the completeness in the recording of pathology and radiology reports, an audit on the methods of lymph node retrieval, and clinican surveys assessing the

For peer review only - http://bmjopen.bmj.com/site/about/guidelines.xhtml

management of patients in the oncological, surgical and aeaethetics settings. All clinicians who are members of regional MDTs as part of the discipline being assessed will be eligible to participate.

Data collection

Existing population-based datasets

The YCR BCIP seeks to make maximum use of routine NHS datasets and these are brought together in the UK Colorectal Cancer Intelligence Hub[18] where data from the NCRAS are linked to other datasets relevant to colorectal cancer to provide the richest data possible and enable analysis of the full cancer pathway. These include, but are not limited to: Hospital Episode Statistics (HES), Radiotherapy Dataset, Systematic Anti-Cancer Therapy Datatset and Routes to Diagnosis. To provide a baseline, this data will cover the period from 1st January 2005 until the start of the programme and be routinely collected until the end of the study to assess changes throughout it.

All patients in the NCRAS data will be assigned a MDT using the HES procedure closest to the patient's diagnosis date. If no procedure is found, the closest inpatient or outpatient appointment to the diagnosis date at a hospital with a colorectal MDT is used. Those not assigned a MDT (<1% of patients) will be excluded from analyses. The assigned MDT will be assumed to have been responsible for the patient's management and treatment options.

Direct PROMs and tissue collection

This research project is adopted by the NIHR Yorkshire and Humber Clinical Research Network and therefore recruitment will be undertaken across the region by network research and clinical staff working collaboratively. Recruitment will run over a 30 month period.

Eligible patients will be identified and approached in two ways.

1. Identified via the MDT and informed about the study by consultant letter sent out with their appointment letter for the primary pre-assessment clinic visit . Where possible, patients will be approached about the study at this clinic appointment. Patients missed at this appointment will be contacted at the earliest convenient time point and informed about the study.

For peer review only - http://bmjopen.bmj.com/site/about/guidelines.xhtml

2. Identified by their NHS clinical team if they present as an acute admission (for example with a bowel obstruction) and informed about the study by their clinical team following the emergency intervention (e.g. surgery).

Participants will have the option of completing a PROMs questionnaire either online (ePROMs) using the University of Leeds secure questionnaire administration system QTool [19], accessed via the study website, (www.YCRBCIP.leeds.ac.uk) or on paper (pPROMs) with a pre-paid return envelope. They will be asked to complete the questionnaire as soon as possible after consenting. This may be completed while attending hospital at the time of consent or later at home. Just prior to the 12 month follow-up questionnaire the patient status will be checked via NCRAS to confirm that the patient is still alive. Patients will then be sent an email/letter 12 months post-diagnosis by the YCR BCIP research team, inviting them to complete PROMs again (on paper or online according to patient preference). At both time points reminder letters or emails will be sent at two weeks and followed up two weeks later (if no response) with the questionnaire being resent with the reminder letter. The process is outlined in Figure 2.

Participants will be asked to complete a range of validated generic, cancer-specific and colorectal cancer-specific PROMs in addition to socio-demographic characteristics. Content has been informed by: clinical relevance, opinion of service users, overall length and participant burden[20], reviews of questionnaires measuring quality of life in colorectal cancer patients[21-25] and UK recommendations for the core outcomes set for trials in colorectal cancer surgery[26].

The questionnaire comprises four sections at baseline and five sections at follow-up. The questionnaire is estimated to take 30-35 minutes to complete.

Your overall health and quality of life (both time points)

- European Organisation for Research and Treatment of Cancer (EORTC) Quality of Life Questionnaire QLQ-C30, the colorectal cancer specific module and items from the prostate, endometrial and cervical cancer modules [27-31]
- EuroQol Group EQ-5D-5L [32]

Your everyday life and well-being (both time points)

- Social Difficulties Inventory (SDI-21) [33 34]
- The Short Warwick-Edinburgh Mental Well-being Scale (SWEMWBS) [35 36]

For peer review only - http://bmjopen.bmj.com/site/about/guidelines.xhtml

Manging your health (both time points)

- Self-efficacy for Managing Chronic Disease [37]
- The Brief Illness Perceptions Questionnaire (B-IPQ) [38]

Questions about you (both time points)

- Self-reported socio-demographic and clinical details

The financial cost of cancer (second time point only)

- A questionnaire developed in-house based on one used in the ePOCS study[39].

The tissue samples to be collected for this study are:

1. excess pre-treatment, diagnostic biopsy tumour tissue
2. excess tissue following surgical resection (tumour and tumour-associated normal mucosal tissue)
3. excess tumour tissue following the biopsy or resection of distant metastases

The tissue samples will be formalin-fixed and paraffin wax-embedded as per the routine histopathology procedures at the patients' hospital. Tissue blocks will be used for any routine clinical diagnostic procedures required by the hospital before being sent to Pathology at the University of Leeds, and will be available to return to the hospital for further clinical testing if required.

Based on the number of diagnoses per year, there is potential to consent approximately 8,250 patients from sites across the region over a 30 month recruitment period. However due to eligibility restrictions, consent by MDT and by individuals, administrative/practical issues and staggered site recruitment start dates, the participation rate is likely to be less. Using estimates based on previous studies [40 41] the consent rate is estimated to be around 42% and the an attrition rate of approximately 35% at the 12 months follow-up point, giving a sample size of at least 1200. This will provide MDTs with a comprehensive descriptive profile of their patients in terms of quality of life outcomes; more complex analyses will be undertaken if sufficient numbers are accrued.

Audit and survey data

Audit data will be limited to data that are collected as part of routine patient care. For example, anonymised MRI scans that have been performed as a part of patient care may be used as part of an educational training initiative. No specific recruitment will be performed for these data as only anonymised data used as part of routine patient care will be used.

For peer review only - http://bmjopen.bmj.com/site/about/guidelines.xhtml

Clinicians at regional MDTs will complete surveys anonymously online using www.onlinesurveys.ac.uk (formally Bristol Online Survey). These surveys will be used to assess clinical practice and patient management. For example, a survey regarding the use of adjuvant chemotherapy will be used to compare regional practice to the most recent evidence basis and used to inform a regional guideline for the treatment of these patients. Regional MDT clinicians will be invited to complete the online surveys via email and through speciality group meetings.

Consent

Existing population-based datasets

The data is derived from patient-level information collected by the NHS, as part of the care and support of cancer patients. NCRAS has specific legal permission to collect this information without the need to seek consent, however, patients can ask NCRAS to remove their details from the cancer registry at any time. Access to cancer registration data and the other routine health datasets used in this study is controlled by the Public Health England (PHE) Office for Data Release, and is only approved for permitted medical purposes[17]. This work is covered by a data sharing contract with PHE (ODR1516_369).

Direct PROMs and tissue collection

Identified patients will be provided with full study information (written and verbal) by specialist research nurses or Clinical Nurse Specialists who have undergone Good Clinical Practice (GCP training) to assure the rights, safety and wellbeing of research participants are protected[42]. Written consent may be taken at the time of this approach but patients will be given up to a week to think about study participation. Patients who wish to join the study will be asked to read, complete and sign a consent form, including their contact details name, address and/or email address. The person taking consent will also record the patient's date of birth, NHS number and gender. Patients must consent to both PROMs participation and tissue collection to be included in the study.

Patients are asked to consent to their clinical team being informed in the event that clinically relevant laboratory results are found. This includes the possibility of hereditary conditions identified through tumour testing "e.g., Lynch syndrome or deficient mismatch repair status". These will be confirmed through routine NHS Clinical Genetics testing after

For peer review only - http://bmjopen.bmj.com/site/about/guidelines.xhtml

counselling following referral from the local clinical teams with no germline testing taking place through the programme.

Audit and survey data

No specific consent will be needed for these data as only anonymised data used as part of routine patient care will be used, for example specimen photographs or scans will have all identifiers removed. Consent for the surveys will be implied when the clinician completes a survey that they have been invited to.

Data Linkage

Whilst intitally the existing population datasets and direct PROMs and tissue collection will be analysed separately, these will subsequently be linked together through the UK Colorectal Cancer Intelligence Hub using name, NHS number and date of birth. This will provide additional patient characterisitcs for analysis of the PROMs and tissue data.

Analysis

Existing population-based datasets

Baseline assessments of care in the region are to be performed on individuals diagnosed with colorectal cancer in Yorkshire and Humber, enabling comparison between teams in the region and with national data[16]. Initially this includes using descriptive analysis and statistical methods such as regression modelling, survival analysis and funnel plots [43] comparing the following data: demographic characteristics, tumour characteristics, surgery and oncology management and short and long-term outcomes.

Some analyses will be rerun periodically over the course of the programme to evaluate the impact on outcomes of specific educational interventions. The measures to be analysed and the sources of these can be found in Table 1.

Direct PROMs and tissue collection

Descriptive statistics will be used to report the questionnaire results and assess the quality of life outcomes of the participants. Following data linkage, the outcomes will be analysed according to stage of disease, treatment type, comorbidity, age, ethnic and sociodemographic group (and other relevant variables). These descriptive analyses will identify potential relationships of interest which can be investigated further. Regression modelling will be used to investigate associations amongst the different types of variables to identify statistically and clinically significant risk factors and predictors of outcomes. In order

For peer review only - http://bmjopen.bmj.com/site/about/guidelines.xhtml

to be robust, analyses will require appropriate adjustment for case-mix and other confounding factors and may require more complex techniques, such as the modelling of hierarchies within the data (multilevel modelling), post-hoc weighting to overcome response bias and multiple imputation of missing data.

Tissue samples removed at surgery or biopsy which are surplus to routine clinical requirements will be utilised by the research team for upfront testing of novel biomarkers. YCR BCIP will test for novel or rare potentially treatable targets via a range of molecular techniques. The immunohistochemical markers of interest include, HER2, PTEN, PD-1 and PD-L1, amphiregulin and epiregulin, and immune system markers CD3 and CD8. The study will also undertake phenotype analysis by using high-resolution scanned images of tumours using novel algorithms to identify improved prognostic and predictive markers of outcome. Next Generation Sequencing and/or pyrosequencing will be performed on any extracted DNA to identify tumours with specific gene hotspot mutations including, KRAS, NRAS, BRAF, EGFR and PIK3CA. Other molecular markers linked to causation, outcome and response to therapy will also be investigated e.g. bacterial toxin carriage or fusobacterium nucleatum. Analysis of RNA expression levels of receptor ligands such as amphiregulin and epiregulin, and HER2 and HER3 may also be performed. The biomarkers will be linked to patient data (population-based and PROMs) and undergo regression modelling to identify associations to understand how tumour biology influences outcomes, response and quality of life and how tumour biology can be influenced by lifestyle.

Audit and survey data

The analyses of audit and survery data will be dependent clinical specialities involved and the nature of the data collected. For example, the completion of MRI scans for rectal cancer at an educational training initiative would be assessed by agreement cofficients and results of surveys will be analysed using descriptive statistics.

Data Safeguards

Participant recruitment will be undertaken by each Trust involved in the study. Each research network site will allocate a study ID for potential participants. They will use a University of Leeds secure electronic transfer system every two weeks to inform the YCR BCIP research team of all recruitment activity. This will include the consented patients contact details, date of birth and NHS number to allow for follow-up and to ensure tissue

For peer review only - http://bmjopen.bmj.com/site/about/guidelines.xhtml

blocks are appropriately labelled for tracking and data linkage. All subsequent participant contact will be undertaken by the central YCR BCIP team.

Storage of all hard copy documents will be in locked metal filing cabinets in research offices of the University of Leeds with secure access building controls. Tissue samples will be used and stored in a scure building with restricted access. Electronic data with pseudonomysed (allocated ID number) patient information will be stored in a secure environment. These files will only be accessible to relevant members of the study analysis team. Where temporary storage of sensitive data is required (e.g. contact details for sending out repeat questionnaires), files will be accessible only to relevant members of the research team and not stored with any linked data. Members of the analysis team will not have access to any identifiable data. Hard copy data will be kept for 5 years following the end of the study (until 2026) for long term follow-up.

Development of Educational Interventions

The analyses will be disseminated and discussed by regional MDTs at events for each clinical discipline. Agreements of educational needs to improve care will be agreed along with any additional data capture and audit processes that teams agree are required. The process is repeated with ongoing data analyses to establish the improvements in management and outcomes that have occurred (Figure 1). As YCR BCIP progresses, further work will be undertaken looking at other outcomes including, but not limited to, those related to screening, pathology and long-term outcomes.

Public Patient Involvement

Patients and carers were actively engaged through the PROMS Working Group to develop the design and content of the patient questionnaires, the patient information sheet and consent form. At the request of the patients and carers, additional questions were included around the financial impact of cancer and a specific request was made to EORTC to amend the EORTC colorectal module and add specific questions from other EORTC modules to understand side effects of cancer and cancer treatments. EORTC granted these specific amendments for this study. The electronic and paper copies of the final draft questionnaires were tested with patients attending a colorectal cancer follow-up clinic at one of the region hospitals. Modification to the layout of the questionnaires were made following the results of the testing. The testing gave an understanding of the length of time

For peer review only - http://bmjopen.bmj.com/site/about/guidelines.xhtml

it took patients to complete the questionnaires. The PROMS Working Group will remain active throughout the length of the study; the group will be kept apprised of recruitment levels and early results. It is expected that the patients will advise on the analysis and how the results are communicated to the regional clinical teams and wider audiences.

Discussion

The YCR BCIP study ambitiously aims to improve outcomes for patients with colorectal cancer across the Yorkshire and Humber region by in-depth analysis of existing and newly captured data, and by actively engaging local MDTs. In its initial stages, this will be done by demonstrating the variation in the demographics, management and outcomes in the region using routine NHS datasets. However, given the limitations of what can be achieved with existing data, the YCR BCIP is collecting additional data to analyse alongside this with the purpose to better understand what is driving the observed variation.

The PROMs data will enrich other study data and allow for an 'in-depth' description of what life is really like for colorectal cancer patients at diagnosis and a year later. At present, the patient voice is not 'measured' routinely as part of clinical practice, neglecting the impact of illness and treatment on the everyday lives of patients. YCR BCIP will change this with the integration of PROMs administration into clinical practice[41]. The PROMs used in this study have been selected with input from experts in colorectal cancer and psychosocial care: patients, clinical nurse specialists and doctors. Although the length of the questionnaire would not be feasible to administer in everyday practice, information on which PROMs provide the most meaningful data will be obtained. In the future risk-stratified follow-up may incorporate not only clinical indicators but also key quality of life indicators to inform best supportive care[44].

Results from the tissue collection and testing could impact on treatment and follow-up decisions for the participating patients and, potentially, their families. For example, the results may indicate that a patient could benefit from a targeted treatment being tested through an open clinical trial if they develop an indication for further treatment e.g. Medical Research Council FOCUS4 [45]. Increased risks of having a hereditary condition may be identified, which not only has implications for the patient but also their family.

For peer review only - http://bmjopen.bmj.com/site/about/guidelines.xhtml

The YCR BCIP is offering a novel approach to address the variation in colorectal cancer care across a large region of the UK. Engagement of the region's MDTs with their data will lead to a range of educational initiatives, studies and clinical audits that aim to optimise practice across the region. It is planned that the outcomes of these will be presented to the relevant specialty group for review and to develop actions based on findings. The main limiting factor for the success of the study is that to understand the overall picture of colorectal cancer care and the ability to improve this in the region, relies on the extent of engagement from MDTs.

Ethics and dissemination

The study was granted ethical approval (17/WM/0374) by the West Midlands – Solihull Research Ethics Committee in December 2017. The study was approved by the Health Research Authority and granted approval for inclusion in the National Institute for Health Research's portfolio of studies in December 2017 (Project ID 227673). Further work from the seven specialty groups involved in the programme: surgery, radiology, pathology, clinical oncology, medical oncology, nursing and anaesthetics, will be reported as publications soon.

Authors' contributions

PQ is the principal investigator.NW, PW and EM are coinvestigators and/or workstream leads. Together these authors conceived and designed the study. HR and JM manage the study. JT provides statistical support. AG provides pathology support. All authors contributed to writing of the manuscript and have approved a final version.

Competing interests

The authors declare that they have no competing interests.

Funding

This work was funded by Yorkshire Cancer Research (Award reference number L394). Prof Quirke holds an NIHR Senior Investigator award. The UK Colorectal Cancer Intelligence Hub is supported by Cancer Research UK (C23434/A23706).

Acknowledgements

For peer review only - http://bmjopen.bmj.com/site/about/guidelines.xhtml

The YCR BCIP study group includes Philip Quirke, Paul Finan, Penny Wright, Nicholas West, Matthew Seymour, Eva Morris, David Sebag-Montefoire, Daniel Swinson, Damian Tolan, Simon Howell, Peter Brown, John Taylor, Amy Glover, Aidan Hindley, Hannah Rossington, Jackie Mara, Emily Boldison. The YCR BCIP research team acknowledges the support of the National Institute for Health Research Clinical Research Network (NIHR CRN).

References

1. Allemani C, Matsuda T, Di Carlo V, et al. Global surveillance of trends in cancer survival 2000–14 (CONCORD-3): analysis of individual records for 37 513 025 patients diagnosed with one of 18 cancers from 322 population-based registries in 71 countries. *The Lancet* 2018 doi: 10.1016/s0140-6736(17)33326-3
2. Heald RJ, Ryall RD. Recurrence and survival after total mesorectal excision for rectal cancer. *Lancet* 1986;1(8496):1479-82. [published Online First: 1986/06/28]
3. Iversen LH, Green A, Ingeholm P, et al. Improved survival of colorectal cancer in Denmark during 2001-2012 - The efforts of several national initiatives. *Acta Oncol* 2016;55 Suppl 2:10-23. doi: 10.3109/0284186X.2015.1131331
4. Holm T, Johansson H, Cedermark B, et al. Influence of hospital- and surgeon-related factors on outcome after treatment of rectal cancer with or without preoperative radiotherapy. *Br J Surg* 1997;84(5):657-63.
5. Wibe AM, B.; Norstein, J.; Carlsen, E.; Wiig, J.N.; Heald, R.J.; Langmark, F.; Myrvold, H.E.; Soreide, O.; for the Norwegian Rectal Cancer Group. A National Strategic Change in Treatment Policy for Rectal Cancer - Implementation of Total Mesorectal Excision as Routine Treatment in Norway. A National Audit. *Dis Colon Rectum* 2002;45(7):857-66. doi: 10.1097/01.Dcr.0000019492.53207.70
6. Iversen LH, Ingeholm P, Gogenur I, et al. Major reduction in 30-day mortality after elective colorectal cancer surgery: a nationwide population-based study in Denmark 2001-2011. *Ann Surg Oncol* 2014;21(7):2267-73. doi: 10.1245/s10434-014-3596-7
7. Martling A, Holm T, Rutqvist LE, et al. Impact of a surgical training programme on rectal cancer outcomes in Stockholm. *Br J Surg* 2005;92(2):225-9. doi: 10.1002/bjs.4834
8. Martling AL, Holm T, Rutqvist LE, et al. Effect of a surgical training programme on outcome of rectal cancer in the County of Stockholm. *The Lancet* 2000;356(9224):93-96. doi: 10.1016/s0140-6736(00)02469-7
9. Moran BJ, Holm T, Brannagan G, et al. The English national low rectal cancer development programme: key messages and future perspectives. *Colorectal Dis* 2014;16(3):173-8. doi: 10.1111/codi.12501
10. Morris EJ, Finan PJ, Spencer K, et al. Wide Variation in the Use of Radiotherapy in the Management of Surgically Treated Rectal Cancer Across the English National Health Service. *Clin Oncol (R Coll Radiol)* 2016;28(8):522-31. doi: 10.1016/j.clon.2016.02.002
11. Vallance AE, van der Meulen J, Kuryba A, et al. The timing of liver resection in patients with colorectal cancer and synchronous liver metastases: a population-based study of current practice and survival. *Colorectal Dis* 2018;20(6):486-95. doi: 10.1111/codi.14019
12. Allemani C, Matsuda T, Di Carlo V, et al. Global surveillance of trends in cancer survival 2000-14 (CONCORD-3): analysis of individual records for 37 513 025 patients

For peer review only - http://bmjopen.bmj.com/site/about/guidelines.xhtml

diagnosed with one of 18 cancers from 322 population-based registries in 71 countries. *Lancet* 2018 doi: 10.1016/S0140-6736(17)33326-3
13. YCR. Yorkshire Cancer Research; [Available from: https://yorkshirecancerresearch.org.uk/.
14. Jessop J, Beagley C, Heald RJ. The Pelican Cancer Foundation and The English National MDT-TME Development Programme. *Colorectal Dis* 2006;8 Suppl 3:1-2. doi: 10.1111/j.1463-1318.2006.01060.x
15. Wibe A, Moller B, Norstein J, et al. A national strategic change in treatment policy for rectal cancer--implementation of total mesorectal excision as routine treatment in Norway. A national audit. *Dis Colon Rectum* 2002;45(7):857-66. doi: 10.1007/s10350-004-6317-7
16. Abstracts of the Association of Coloproctology of Great Britain and Ireland Annual Meeting, 3-5 July 2017, Bournemouth, UK. *Colorectal Dis* 2017;19 Suppl 4:3-66. doi: 10.1111/codi.13849
17. NCRAS. National Cancer Registration and Analysis Service: Public Health England; [Available from: https://www.gov.uk/guidance/national-cancer-registration-and-analysis-service-ncras.
18. BCI-UK. UK Colorectal Intelligence Hub, University of Leeds; [Available from: https://bci.leeds.ac.uk/.
19. Ashley L, Jones H, Thomas J, et al. Integrating cancer survivors' experiences into UK cancer registries: design and development of the ePOCS system (electronic Patient-reported Outcomes from Cancer Survivors). *Br J Cancer* 2011;105 Suppl 1:S74-81. doi: 10.1038/bjc.2011.424
20. Foster C, Haviland J, Winter J, et al. Pre-Surgery Depression and Confidence to Manage Problems Predict Recovery Trajectories of Health and Wellbeing in the First Two Years following Colorectal Cancer: Results from the CREW Cohort Study. *Plos One* 2016;11(5) doi: 10.1371/journal.pone.0155434
21. Ganesh V, Agarwal A, Popovic M, et al. Comparison of the FACT-C, EORTC QLQ-CR38, and QLQ-CR29 quality of life questionnaires for patients with colorectal cancer: a literature review. *Supportive Care in Cancer* 2016;24(8):3661-68. doi: 10.1007/s00520-016-3270-7
22. Gilbert A, Sebag-Montefiore D, Davidson S, et al. Use of patient-reported outcomes to measure symptoms and health related quality of life in the clinic. *Gynecologic Oncology* 2015;136(3):429-39. doi: 10.1016/j.ygyno.2014.11.071
23. Hadi M, Gibbons E, Fitzpatrick R. A structured review of patient reported outcome measures for colorectal cancer. In: Patient Reported Outcome Measurement Group UoO, ed. Patient Reported Outcome Measures. Oxford, 2010.
24. McNair AGK, Whistance RN, Forsythe RO, et al. Synthesis and summary of patient-reported outcome measures to inform the development of a core outcome set in colorectal cancer surgery. *Colorectal Dis* 2015;17(11):O217-O29. doi: 10.1111/codi.13021
25. Wong CKH, Chen J, Yu CLY, et al. Systematic review recommends the European Organization for Research and Treatment of Cancer colorectal cancer-specific module for measuring quality of life in colorectal cancer patients. *Journal of Clinical Epidemiology* 2015;68(3):266-78. doi: 10.1016/j.jclinepi.2014.09.021

For peer review only - http://bmjopen.bmj.com/site/about/guidelines.xhtml

26. McNair AG, Whistance RN, Forsythe RO, et al. Core Outcomes for Colorectal Cancer Surgery: A Consensus Study. *PLoS Med* 2016;13(8):e1002071. doi: 10.1371/journal.pmed.1002071
27. Aaronson NK, Ahmedzai S, Bergman B, et al. The European Organization for Research and Treatment of Cancer QLQ-C30: a quality-of-life instrument for use in international clinical trials in oncology. *Journal of the National Cancer Institute* 1993;85(5):365-76.
28. Greimel E, Nordin A, Lanceley A, et al. Psychometric validation of the European Organisation for Research and Treatment of Cancer Quality of Life Questionnaire-Endometrial Cancer Module (EORTC QLQ-EN24). *Eur J Cancer* 2011;47(2):183-90. doi: 10.1016/j.ejca.2010.08.014
29. Greimel ER, Kuljanic Vlasic K, Waldenstrom AC, et al. The European Organization for Research and Treatment of Cancer (EORTC) Quality-of-Life questionnaire cervical cancer module: EORTC QLQ-CX24. *Cancer* 2006;107(8):1812-22. doi: 10.1002/cncr.22217
30. van Andel G, Bottomley A, Fossa SD, et al. An international field study of the EORTC QLQ-PR25: a questionnaire for assessing the health-related quality of life of patients with prostate cancer. *Eur J Cancer* 2008;44(16):2418-24. doi: 10.1016/j.ejca.2008.07.030
31. Whistance RN, Conroy T, Chie W, et al. Clinical and psychometric validation of the EORTC QLQ-CR29 questionnaire module to assess health-related quality of life in patients with colorectal cancer. *Eur J Cancer* 2009;45(17):3017-26. doi: 10.1016/j.ejca.2009.08.014
32. Herdman M, Gudex C, Lloyd A, et al. Development and preliminary testing of the new five-level version of EQ-5D (EQ-5D-5L). *Qual Life Res* 2011;20(10):1727-36. doi: 10.1007/s11136-011-9903-x
33. Wright EP, Kiely M, Johnston C, et al. Development and evaluation of an instrument to assess social difficulties in routine oncology practice. *Qual Life Res* 2005;14(2):373-86.
34. Wright P, Smith AB, Keding A, et al. The Social Difficulties Inventory (SDI): development of subscales and scoring guidance for staff. *Psychooncology* 2011;20(1):36-43. doi: 10.1002/pon.1705
35. Tennant R, Hiller L, Fishwick R, et al. The Warwick-Edinburgh Mental Well-being Scale (WEMWBS): development and UK validation. *Health Qual Life Outcomes* 2007;5:63. doi: 10.1186/1477-7525-5-63
36. Bartram DJ, Sinclair JM, Baldwin DS. Further validation of the Warwick-Edinburgh Mental Well-being Scale (WEMWBS) in the UK veterinary profession: Rasch analysis. *Qual Life Res* 2013;22(2):379-91. doi: 10.1007/s11136-012-0144-4
37. Lorig KR, Sobel DS, Ritter PL, et al. Effect of a self-management program on patients with chronic disease. *Eff Clin Pract* 2001;4(6):256-62.
38. Broadbent E, Petrie KJ, Main J, et al. The brief illness perception questionnaire. *J Psychosom Res* 2006;60(6):631-7. doi: 10.1016/j.jpsychores.2005.10.020
39. Marti J, Hall PS, Hamilton P, et al. The economic burden of cancer in the UK: a study of survivors treated with curative intent. *Psychooncology* 2016;25(1):77-83. doi: 10.1002/pon.3877
40. Ashley L, Jones H, Thomas J, et al. Integrating patient reported outcomes with clinical cancer registry data: a feasibility study of the electronic Patient-Reported Outcomes

For peer review only - http://bmjopen.bmj.com/site/about/guidelines.xhtml

From Cancer Survivors (ePOCS) system. *J Med Internet Res* 2013;15(10):e230. doi: 10.2196/jmir.2764
41. Black N, Burke L, Forrest CB, et al. Patient-reported outcomes: pathways to better health, better services, and better societies. *Qual Life Res* 2016;25(5):1103-12. doi: 10.1007/s11136-015-1168-3
42. NIHR. National Institute for Health Research; [Available from: https://www.nihr.ac.uk/health-and-care-professionals/learning-and-support/good-clinical-practice.htm.
43. Spiegelhalter DJ. Funnel plots for comparing institutional performance. *Stat Med* 2005;24(8):1185-202. doi: 10.1002/sim.1970
44. Watson EK, Rose PW, Neal RD, et al. Personalised cancer follow-up: risk stratification, needs assessment or both? *Br J Cancer* 2012;106(1):1-5. doi: 10.1038/bjc.2011.535
45. Kaplan R, Maughan T, Crook A, et al. Evaluating many treatments and biomarkers in oncology: a new design. *J Clin Oncol* 2013;31(36):4562-8. doi: 10.1200/JCO.2013.50.7905

Figure Legends

Figure 1: Yorkshire Cancer Research Bowel Cancer Improvement Programme (YCRBCIP) process and study design.

Figure 2: Process for the Patient Reported Outcomes Measures (PROMs) study.

For peer review only

For peer review only - http://bmjopen.bmj.com/site/about/guidelines.xhtml

	Data Source		
	**Existing population-based datasets**	**Direct PROMs and tissue collection**	**Audit and survey data**
**Patient and Tumour Characteristics**			
Age & sex	NCRAS	PROMs	
Ethnicity	NCRAS	PROMs	
Height & Weight		PROMs	
Comorbidity	NCRAS	PROMs	
Socio-economic status	NCRAS	PROMs	
Stage & site	NCRAS		
Method of admission	NCRAS		
**Treatment Variation**			
Surgical resection rate	HES		
Quality of surgery			Audit
Abdominoperineal excision rate	HES		
Use of adjuvant & palliative chemotherapy	SACT		Survey
Use of neoadjuvant radiotherapy	RTDS		
Use of laparoscopic surgery	HES		
Emergency care procedures	HES		Survey
Practice of anaesthetics			Survey
Quality of MRI reporting			Audit
Quality of CT imaging			Audit
Liver metastases resection rate	HES		
Nodal yields and retrieval methods	NCRAS		Audit
**Outcomes**			
30-day Postoperative mortality	NCRAS		
1 to 5 year overall and net survival	NCRAS		
18-month postoperative stoma rate	HES		
Postoperative hospital stay	HES		
Emergency readmission rates	HES		
Overall health and quality of life		PROMs	
Everyday life and well-being		PROMs	
Self-efficacy for Managing Chronic Disease		PROMs	
Financial cost of cancer		PROMs	
Urinary function and faecal incontinence		PROMs	
Sexual functioning		PROMs	
Lower anterior resection syndrome		PROMs	
Molecular subtyping		Tissue	

Abbreviations: Magnetic resonance imaging (MRI), Computerized Tomography (CT), National Cancer Registration and Analysis Service (NCRAS), Routes to Diagnosis (RtD), Hospital Episode Statistics (HES), Systemic Anti-Cancer Therapy Dataset (SACT), Radiotherapy Dataset (RTDS).

Table 1. Measures of patient characteristics, treatment measures and outcomes to analysed and the corresponding data source.

For peer review only - http://bmjopen.bmj.com/site/about/guidelines.xhtml

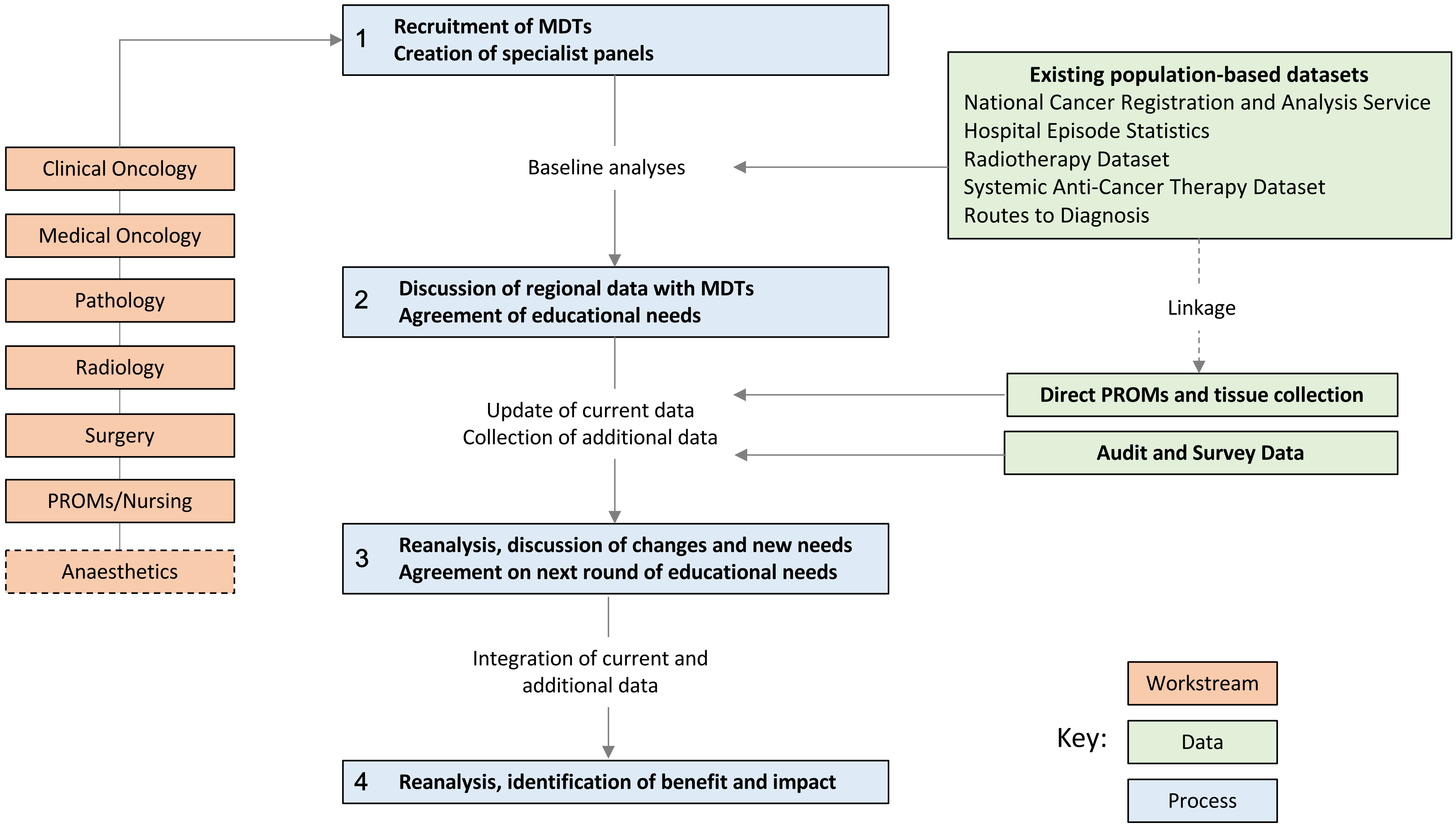
1 Recruitment of MDTs
Creation of specialist panels
Baseline analyses
2 Discussion of regional data with MDTs
Agreement of educational needs
Update of current data
Collection of additional data
3 Reanalysis, discussion of changes and new needs
Agreement on next round of educational needs
Integration of current and additional data
4 Reanalysis, identification of benefit and impact
Clinical Oncology
Medical Oncology
Pathology
Radiology
Surgery
PROMs/Nursing
Anaesthetics
Existing population-based datasets
National Cancer Registration and Analysis Service
Hospital Episode Statistics
Radiotherapy Dataset
Systemic Anti-Cancer Therapy Dataset
Routes to Diagnosis
Linkage
Direct PROMs and tissue collection
Audit and Survey Data
Key:
Workstream
Data
Process

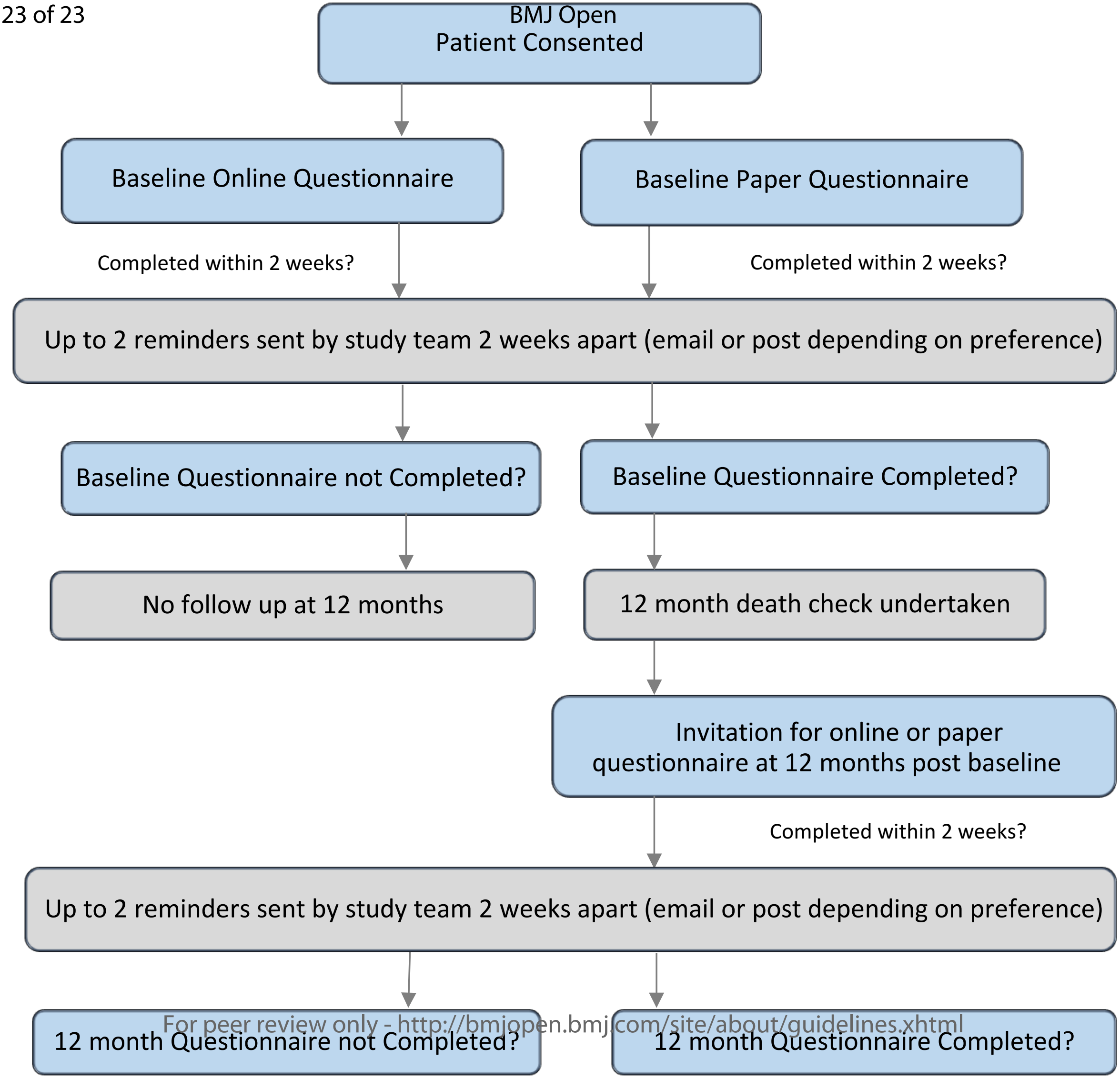

For peer review only - http://bmjopen.bmj.com/site/about/guidelines.xhtml
